# Comparative analysis of cysteine proteases reveals gene family evolution of the group 1 allergens in astigmatic mites

**DOI:** 10.1002/clt2.12324

**Published:** 2023-12-15

**Authors:** Ling Shi, Qing Xiong, Fu Kiu Ao, Tsz Yau Wan, Xiaojun Xiao, Xiaoyu Liu, Baoqing Sun, Anchalee Tungtrongchitr, Ting Fan Leung, Stephen Kwok Wing Tsui

**Affiliations:** ^1^ School of Biomedical Sciences The Chinese University of Hong Kong Hong Kong China; ^2^ Hong Kong Bioinformatics Centre The Chinese University of Hong Kong Hong Kong China; ^3^ Department of Health Technology and Informatics The Hong Kong Polytechnic University Hong Kong China; ^4^ Shenzhen Key Laboratory of Allergy and Immunology, School of Medicine Shenzhen University Shenzhen China; ^5^ State Key Laboratory of Respiratory Disease The First Affiliated Hospital of Guangzhou Medical University Guangzhou China; ^6^ Department of Parasitology, Faculty of Medicine Siriraj Hospital Mahidol University Bangkok Thailand; ^7^ Department of Paediatrics The Chinese University of Hong Kong Hong Kong China; ^8^ Centre for Microbial Genomics and Proteomics The Chinese University of Hong Kong Hong Kong China

**Keywords:** comparative analysis, cysteine protease evolution, gene decay, group 1 allergen homologs, tandem duplication

## Abstract

**Background:**

Astigmatic mites contain potent allergens that can trigger IgE‐mediated immune responses, leading to allergic diseases such as asthma, allergic rhinitis and atopic dermatitis. In house dust mites *Dermatophagoides pteronyssinus* and *Dermatophagoides farinae*, group 1 allergens (Der p 1 and Der f 1), characterized as papain‐like cysteine proteases, have been defined as the major allergens that have high prevalence and potency. Previous studies of mite group 1 allergens mainly focused on identification, comparison of sequence and structure, as well as the investigation of cross‐reactivity. To achieve a comprehensive view of mite group 1 allergens, we performed a comparative genomic analysis of all the cysteine proteases in six astigmatic mite species to elucidate the evolutionary relationships of group 1 allergens.

**Methods:**

Based on the high‐quality and annotated genomes, all the cysteine proteases in six astigmatic mite species were identified by sequence homology search. The phylogenetic relationships, gene synteny and expression levels were revealed by bioinformatic tools. The allergenicity of recombinant cysteine proteases was evaluated by enzyme‐linked immunosorbent assay.

**Results:**

Tandem duplication was revealed as the major feature of cysteine protease gene evolution in astigmatic mites. The high IgE‐binding capacity and the significant expression level of the cysteine protease DP_007902.01 suggested its potential as a novel group 1 allergen of *D. pteronyssinus*. In addition, gene decay events were identified in the skin‐burrowing parasitic mite *Sarcoptes scabiei*.

**Conclusion:**

This comprehensive analysis provided insights into the evolution of cysteine proteases, as well as the component‐resolved diagnosis of mite allergies.

## 1 INTRODUCTION

Medically important astigmatic mites, including house dust mites (HDMs) and storage mites, have long been the major cause of human allergies and affect about 1%–2% of the world population.^1^, ^2^ To date, more than 35 allergen groups of astigmatic mites have been approved by the WHO/IUIS allergen nomenclature sub‐committee (http://allergen.org).^3^ Group 1 allergens, featured as cysteine proteases, have been identified as the major allergens of HDMs, *Dermatophagoides* (*D.*) *pteronyssinus* and *Dermatophagoides farinae*.^4^, ^5^ It has been reported that more than 80% of mite‐allergic patients showed IgE positivity to group 1 allergens of HDMs.^6^ Previous studies of group 1 allergens mainly focused on: (1) the identification and characterization of group 1 allergens in a single astigmatic mite species,^7^, ^8^, ^9^ (2) the investigation of sequence homology, structural difference and cross‐reactivity between the major allergens Der p 1 and Der f 1^10^ ^,^ ^11^. The evolution of the cysteine protease gene family in astigmatic mites has not been fully understood.

In this study, we performed a comparative analysis of all the cysteine proteases in six astigmatic mite species based on the high‐quality genomes. The identification of massively tandemly arrayed cysteine protease genes in HDMs and storage mites revealed the rapid evolution of group 1 allergens. The elevated IgE reactivity and the substantial transcript expression of cysteine protease DP_007902.01 indicated its potential as a novel group 1 allergen in *Dermatophagoides pteronyssinus*. Gene decay events observed in the skin‐burrowing parasitic mite *Sarcoptes (S.) scabiei* suggested its parasitic adaptation. These findings shed light on the evolution of the cysteine protease gene family and provided insights into the development of component‐resolved diagnosis (CRD) in mite allergies.

## 2 METHODS

### 2.1 Genome and transcriptome data

The genome and transcriptome data of *D. farinae*, *D. pteronyssinus*, *Blomia tropicalis* and *Tyrophagus putrescentiae* have been reported in our previous study^2^ and deposited in the NCBI database (BioProject accession: PRJNA174061, PRJNA388362, PRJNA702011, PRJNA706095, respectively). The genome and transcriptome sequencing data of *Psoroptes ovis* and *Sarcoptes scabiei* were downloaded from the NCBI database (BioProject accession: PRJNA521406 for *P. ovis*, PRJNA268368, PRJNA598457, PRJNA749654 and PRJNA304361 for *S. scabiei*).

### 2.2 In silico identification of cysteine protease genes

The protein sequences of reference group 1 allergens (Der p 1, Der f 1, Blo t 1 and Tyr p 1) in HDMs and storage mites were downloaded from the WHO/IUIS allergen nomenclature database.^3^ The protein sequence of group 1 allergen in the parasitic mite *P. ovis* (Pso o 1) was retrieved from the UniProt database^12^ (UniProt accession ID: Q1EIQ30). The group 1 allergens and their cysteine protease homologs in the annotated astigmatic genomes were identified by BLASTP (v2.11.0)^13^ search against the reference group 1 allergens with the option “‐evalue 1e‐6 ‐outfmt 6”. The top matched proteins were putative group 1 allergens, and the proteins with the sequence identity no less than ∼30% were considered as cysteine protease homologs. HMMER (v 3.3.2)^14^ search was further employed to validate the results of BLASTP. The profile hidden Markov model (HMM) was established based on the protein sequence alignment of all the reference group allergens by the hmmbuild command. The cysteine protease homologs were detected using the hmmsearch command against the profile HMM model with the default parameters. The transcriptome mapping and the file format conversion were conducted by Hisat2 (v2.2.1)^15^ and SAMtools (v1.12).^16^ All the identified cysteine protease genes were manually curated based on the annotated genomes and mapped transcriptomes and then visualized in Integrative Genomics Viewer (IGV v2.8.13).^17^ Based on the gene synteny information from the genome annotation gff files, the tandemly arrayed genes (TAGs) and proximally arrayed genes (PAGs) were defined with zero spacer genes and less than 10 spacer genes, respectively.

### 2.3 Phylogenetic analysis

The multiple sequence alignment of all cysteine protease gene clusters was performed using CLUSTAL W^18^ and MUSCLE^19^ in MEGA (v7.0.26).^20^ The phylogenetic trees were further constructed based on maximum likelihood (ML) algorithm with JTT (Jones‐Taylor‐Thornton) model and 95% coverage in MEGA (v7.0.26). The reliability of the phylogenetic trees was tested by the bootstrap method with 100 replications. The phylogenetic trees were edited and annotated by the online tool Interactive Tree of Life (iTOL v6).^21^

### 2.4 Subjects and serum samples

This study was approved by the institutional review board of Prince of Wales Hospital (CREC Ref. No.: 2018.663) for using patient sera in enzyme‐linked immunosorbent assay (ELISA) experiments. The subjects and/or their parents have provided informed consent to participate. The serum samples were collected at Prince of Wales Hospital (Hong Kong) from 15 patients with dust mite allergies and 8 healthy individuals as negative controls (Supplementary Table S7).

### 2.5 Cloning, expression, purification and ELISA of recombinant proteins

The CDS and protein sequences of three *D. pteronyssinus* cysteine proteases (DP_002156.02, DP_007902.01 and DP_014036.02) were collected from the genome after manual curation (Supplementary Table S8). The 3D structures of the three cysteine proteases were established by SWISS‐MODEL server^22^ using homology modeling (Supplementary Figure S13). The codon‐optimized CDS sequences for pro‐forms of the three cysteine proteases were synthesized, subcloned into plasmid pET‐30a(+), and expressed in TOP10 *Escherichia coli*. The recombinant proteins were then purified by Ni‐NTA affinity chromatography. The cloning, expression and purification of recombinant proteins were performed by Sangon Biotech (Shanghai, China). The pro‐forms of cysteine proteases were used for IgE ELISA since the pro‐form of Der p 1 has been suggested to be more efficiently immobilized, whilst the IgE epitopes blocked by the pro‐domain in the solution structure of the pro‐form could be exposed and recognized by IgE when immobilized.^23^ The levels of IgE reactivity to the recombinant cysteine proteases were measured using the serum samples of 15 HDM‐sensitized patients and 8 non‐allergic subjects by ELISA. Each serum sample from the HDM‐sensitized subjects was tested in replicate. Each well of the 96‐well microtiter plate was coated with the purified proteins (0.5 μg/well) dissolved in coating buffer (100 mM Na_2_CO_3_ · 100 mM NaHCO_3_, pH = 9.6) and incubated at 37°C for 3 h. The plates were washed with PBST three times and blocked with 8% fetal bovine serum (Gibco) in PBS at room temperature for 2 h. Serum samples were added with 1:5 dilution in blocking buffer and incubated at 4°C overnight. The plates were washed with PBST three times and incubated with HRP‐conjugated anti‐human IgE antibodies (Thermo Fisher Scientific) at 1:1000 dilution at room temperature for 1 h. The plates were then washed with PBST five times and added with TMB substrate. The reaction was stopped with 0.1 M sulfuric acid and the absorbance was measured at 450 nm using a Multiskan GO microplate spectrometer (Thermo Scientific). The cutoff was calculated as the mean absorbance ± 2SD of all the non‐allergic subjects. The statistical significance between each HDM‐sensitized subject and the non‐allergic subjects was determined by Student's *t*‐test.

### 2.6 Quantification of gene expression level

All the coding sequences of *D. farinae*, *D. pteronyssinus*, *B. tropicalis* and *T. putrescentiae* were extracted by GffRead (v 0.12.7)^24^ and indexed by Salmon (v1.5.2).^25^ Two adult mite transcriptome datasets of *D. farinae* (NCBI SRA accession: SRR9005248, SRR9005249), *D. pteronyssinus* (NCBI SRA accession: SRR7617923, SRR7617924), *B. tropicalis* (NCBI SRA accession: SRR13742047, SRR13742048) and *T. putrescentiae* (NCBI SRA accession: SRR13837414, TP1 and TP3) were mapped to the indexed CDS of six astigmatic mites respectively and the gene expression levels of the cysteine protease homologs were examined and represented by transcripts per million using Salmon (v1.5.2). Statistical significance was determined by one‐way ANOVA and Dunnett's multiple comparison test in GraphPad Prism (v9).

### 2.7 Catalytic triad analysis and epitope mapping

The conserved catalytic sequence motifs of cysteine protease in dust mites were obtained from previous studies.^26^, ^27^, ^28^ The linear epitopes of Der f 1 and Der p 1 were retrieved from the Immune Epitope Database.^29^ Multiple sequence alignment of cysteine protease genes was performed using Clustal Omega^30^ from European Bioinformatics Institute.^31^ Based on the multiple sequence alignment results, the catalytic triads were identified, and the epitopes were mapped. The active genes were defined as the maintenance of all the three active catalytic sites.

### 2.8 Selection pressure analysis

The coding sequence alignment of the cysteine protease gene clusters were performed using CLUSTALW and MUSCLE in MEGA (v7.0.26). To examine the selection pressure, the ratios of non‐synonymous to synonymous substitutions (dN/dS) were estimated based on codons by HyPhy (v2.5.31).^32^ The site‐level selection of each gene cluster was tested by two ML approaches, mixed effects model of evolution and fixed effects likelihood.^33^, ^34^ The sites under positive and negative selection were reported with *p* < 0.05.

## 3 RESULTS

### 3.1 Identification of massively tandemly duplicated cysteine proteases

**FIGURE 1:**
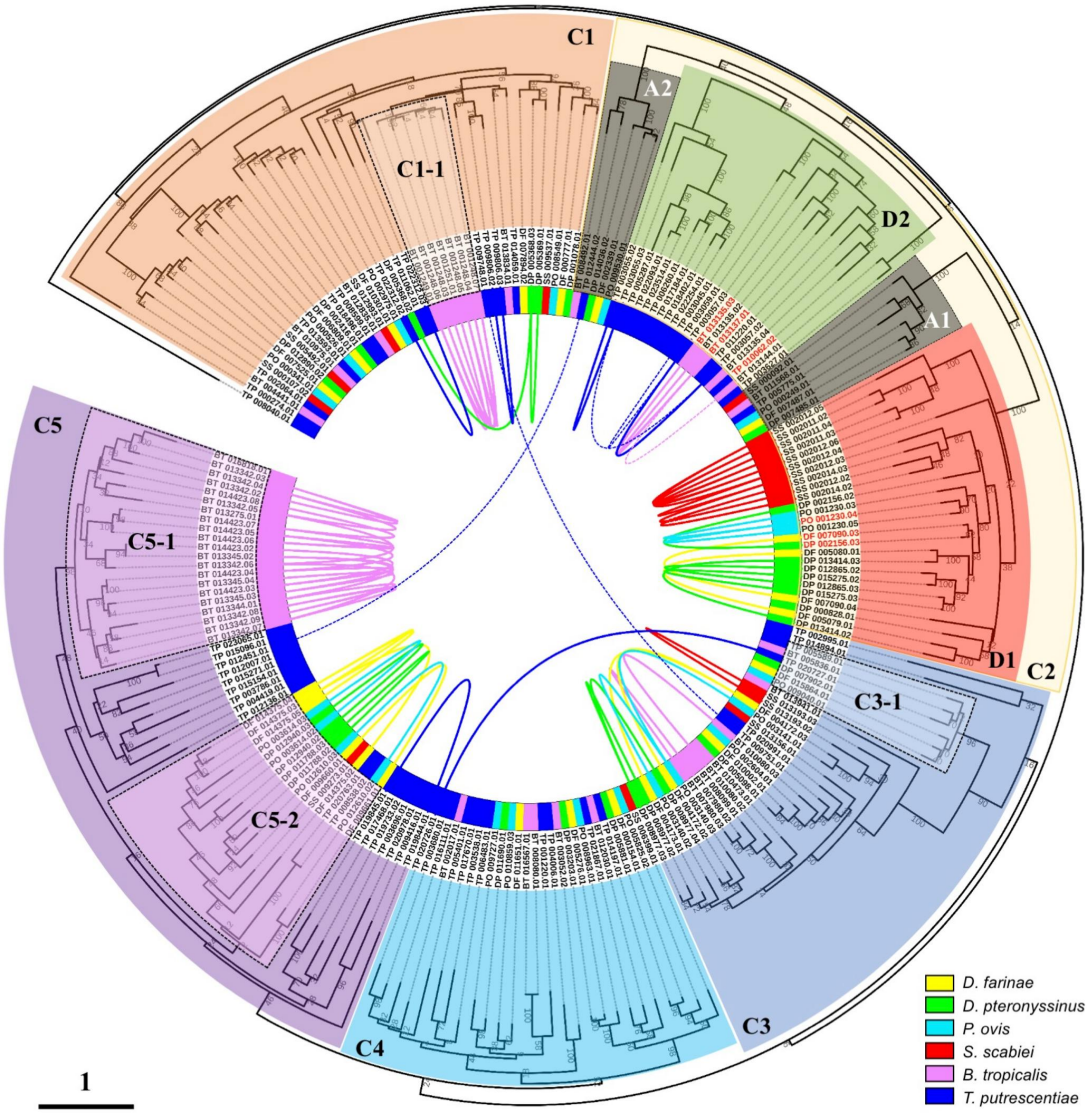
Phylogenetic relationships of cysteine proteases in six astigmatic mites. Except for the outlier TP_008040.01, all the other cysteine proteases were classified into five large clusters (C1‐C5) based on the phylogenetic relationships. Group 1 allergens (Der f 1, Der p 1, Pso o 1, Blo t 1 and Tyr p 1) were marked as red in label. The tandemly/proximally arrayed genes were connected by curved solid lines and curved dashed lines, respectively.

To identify all cysteine proteases, both BLASTP and HMMER searches were performed using the amino acid sequences of group 1 allergens from *D. pteronyssinus* (Der p 1), *D. farinae* (Der f 1), *Blomia (B.) tropicalis* (Blo t 1), and *Tyrophagus* (*T.*) *putrescentiae* (Tyr p 1) against the annotated protein sequences of six mite genomes. A total of 214 cysteine proteases from six astigmatic mites were identified. The number of cysteine proteases in *T. putrescentiae* and *B. tropicalis* was 66 and 52, respectively, which is much higher than that of any other four mite species (Table 1). Based on the phylogenetic relationships, we further classified the genes into five large clusters (C1–C5) except for one outlier from *T. putrescentiae* (TP_008040.01) (Figure 1).

**TABLE 1:** Summary of cysteine protease genes in six astigmatic mite species.

	*Dermatophagoides pteronyssinus*	*Dermatophagoides farinae*	*Psoroptes ovis*	*Sarcoptes scabiei*	*Blomia tropicalis*	*Tyrophagus putrescentiae*	Total
Total	29	25	22	20	52	66	214
Tandemly/proximally arrayed^a^	18	13	10	12	34	20	107
Active^b^	24	22	20	14	44	45	169

^a^ Tandemly arrayed genes were linked without spacer genes, and proximally arrayed genes were separated by less than 10 spacer genes.

^b^ Active genes were defined based on the alignment of catalytic triads (C‐H‐N) in cystine proteases.

Gene duplication has been demonstrated to be an important process for genome evolution. Tandem duplication is one of the major mechanisms contributing to gene family expansion and novel gene generation.^35^ Tandemly or proximally arrayed genes (T/PAGs) are duplicated genes from the same family with zero or a few non‐homologous spacer gene(s) separated.^36^ Given the large number of cysteine proteases identified, the gene duplication events were investigated in the six astigmatic mite genomes. Based on the sequence identity and synteny, 107 genes were either tandemly arrayed without spacer genes or proximally arrayed with a few (<10) spacer genes separated (Table 1). Among all six astigmatic mite species, *B. tropicalis* contained the largest number of T/PAGs, while *Psoroptes* (*P*.) *ovis* had the fewest T/PAGs (Table 1).

### 3.2 Comparative analysis of group 1 allergen cluster in HDMs

**FIGURE 2:**
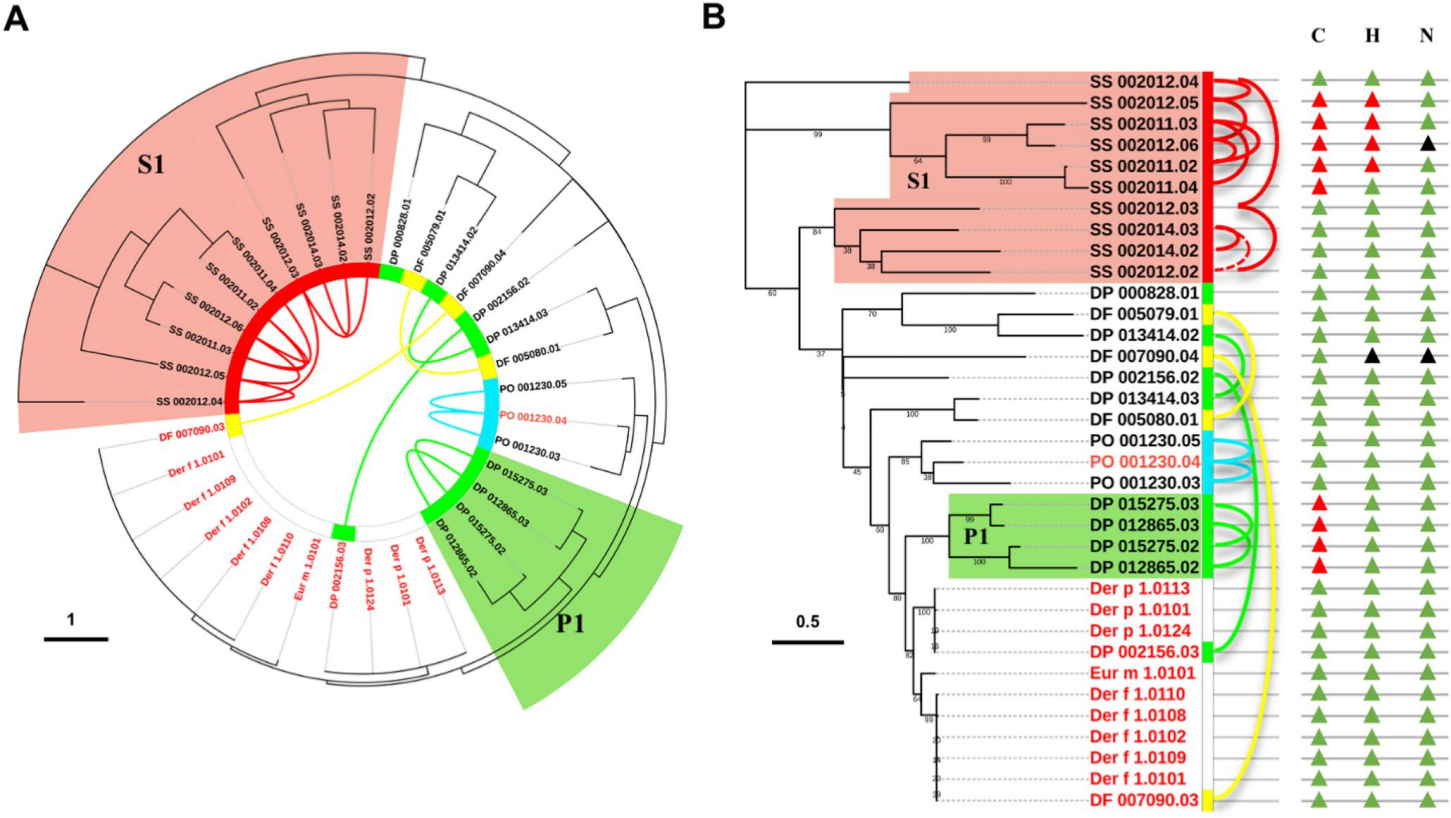
Phylogenetic analysis of D1 cluster and the alignment of catalytic triads. (A) The phylogenetic analysis of D1 cluster genes, with “DP” as *Dermatophagoides pteronyssinus*, “DF” as *Dermatophagoides*, “PO” as *Psoroptes ovis* and “SS” as *Sarcoptes scabiei*. The tandemly/proximally arrayed genes were connected by solid curved lines and dashed curved lines, respectively. The group 1 allergens Der f 1 and Der p 1 were labeled in red. (B) The catalytic triads (C‐H‐N) of cystine protease were aligned, with the active sites, site‐mutated sites, and deleted sites marked in green triangles, red triangles and black triangles, respectively.

D1 was a typical cluster that contained a total of 26 cysteine proteases from *D. pteronyssinus*, *D. farinae*, *P. ovis* and *S. scabiei*, with 25 of them tandemly arrayed. For the T/PAGs, 10 genes were identified in *S. scabiei*, 8 in *D. pteronyssinus*, 4 in *D. farinae* and 3 in *P. ovis*. The phylogenetic analysis of all the cysteine proteases in the D1 cluster together with the reported HDM group 1 allergens (Der p 1, Der f 1, Eur m 1) was performed. In the phylogenetic tree, DP_002156.03 and DF_007090.03 shared identical lineage with Der p 1 and Der f 1, respectively, whilst DF_007090.03 was also closely related to group 1 allergen of HDM *Euroglyphus mayne*i (Eur m 1) (Figure 2A). The sequence identity between DP_002156.03, DF_007090.03 and the reported Der p 1, Der f 1 was higher than 99% (Supplementary Table S1). These findings suggested DP_002156.03 as Der p 1 and DF_007090.03 as Der f 1 in our mite genomes.

Cysteine proteases contain conserved catalytic triads (C‐H‐N) that can indicate the enzyme activity.^28^ Based on the catalytic triad analysis, a total of 16 active cysteine proteases were identified in the D1 cluster (Figure 2B). Interestingly, in the subcluster S1 that contained T/PAGs of *S. scabiei,* 5 of the T/PAGs had site mutations at catalytic sites (Figure 2B). Likewise, in P1, T/PAGs of *D. pteronyssinus* were also inactive due to the cysteine mutations (Figure 2B). Selection pressure analysis identified 7 sites under positive selection and 29 sites under negative selection in *D. pteronyssinus* (Supplementary Table S2A). In *D. farinae*, 2 sites were positively selected, and 18 sites were negatively selected (Supplementary Table S2B). As for cysteine protease genes from *S. scabiei* in the D1 cluster, 2 sites were under positive selection and 57 sites were under negative selection (Supplementary Table S2C).

**FIGURE 3:**
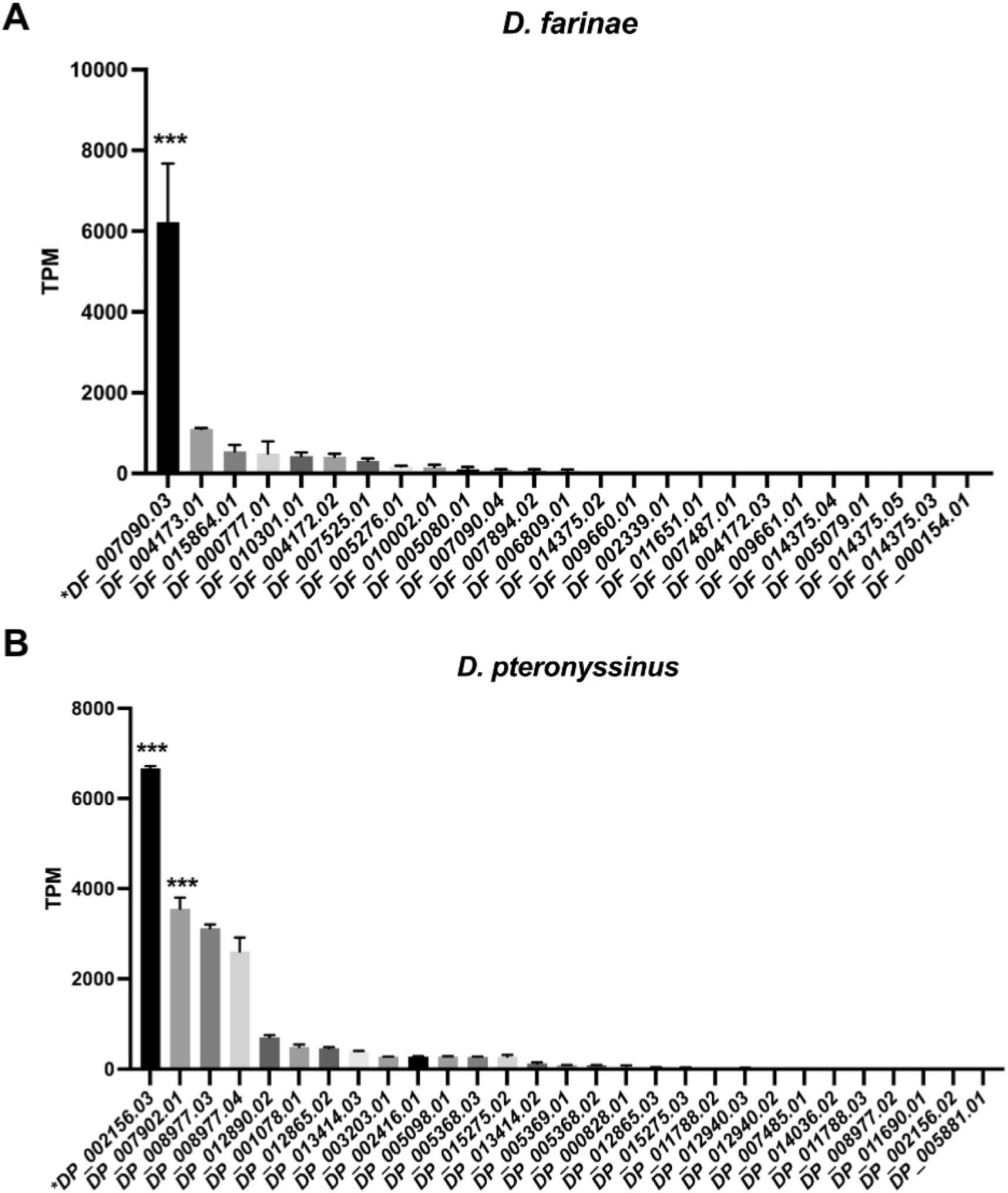
The transcript expression of cysteine proteases in house dust mites. The transcript expression levels of all the cysteine protease genes in (A) *Dermatophagoides farinae* and (B) *Dermatophagoides pteronyssinus* were quantified as transcripts per million (TPM) using two adult mite transcriptome data of *D. farinae* and *D. pteronyssinus*, respectively. Der f 1 (DF_007090.03) and Der p 1 (DP_002156.03) were marked with asterisks. Data were shown as mean ± SD. Statistical significance was determined by one‐way ANOVA, followed by Dunnett's multiple comparison test. ***p < 0.001.

Previously, proteomic identification of group 1 allergens in *D. pteronyssinus* and *D. farinae* were performed using pooled mite‐allergic patient sera.^4^, ^5^ The protein extracts of HDMs were applied to 2D‐PAGE experiment and blot analysis of IgE reactivity, then the peptide sequences of IgE‐binding proteins were identified by MALDI‐TOF mass spectrometry (MS). We collected the peptide sequences of IgE‐binding proteins and mapped them to D1 cluster genes. The results demonstrated that the sequences of IgE‐binding proteins in *D. pteronyssinus* can only be aligned to DP_002156.03 (Supplementary Figure S1). Likewise, the peptide sequences of IgE‐binding proteins determined in *D. farinae* were unique to DF_007090.03 (Supplementary Figure S1). Moreover, the transcript expression levels of DP_002156.03 and DF_007090.03 were significantly higher than those of all the other cysteine protease genes examined (Figure 3). These findings confirmed that DP_002156.03 and DF_007090.03 were Der p 1 and Der f 1, respectively.

In the D1 cluster, we identified several cysteine protease homologs closely related to Der f 1 and Der p 1, all of which exhibited a sequence identity with Der f 1 or Der p 1 greater than 35% (Supplementary Table S3). Although a perfect match of epitope alignment was not observed, multiple conserved and partially conserved sequences of epitopes were identified among Der f 1, Der p 1 and their closely related cysteine protease homologs (Supplementary Figure S2). The allergenicity of DP_002156.02, the tandemly arrayed cysteine protease of Der p 1 (DP_002156.03), was evaluated by ELISA with HDM‐sensitized patient sera. All of the HDM‐sensitized subjects demonstrated significantly elevated IgE sensitivity to the protein extracts of *D. pteronyssinus* (Supplementary Figure S3). Four out of 15 patients had a significantly higher IgE sensitivity to DP_002156.02 than the non‐allergic subjects, resulting in a 26.7% positive rate (Supplementary Figure S4). These findings suggested that the cysteine proteases of *D. pteronyssinus* and *D. farinae* in the D1 cluster might possess potential allergenicity and cross‐reactivity with Der p 1 and Der f 1.

### 3.3 The gene decay of cysteine proteases in the skin‐burrowing parasitic mite *S. scabiei*

**FIGURE 4:**
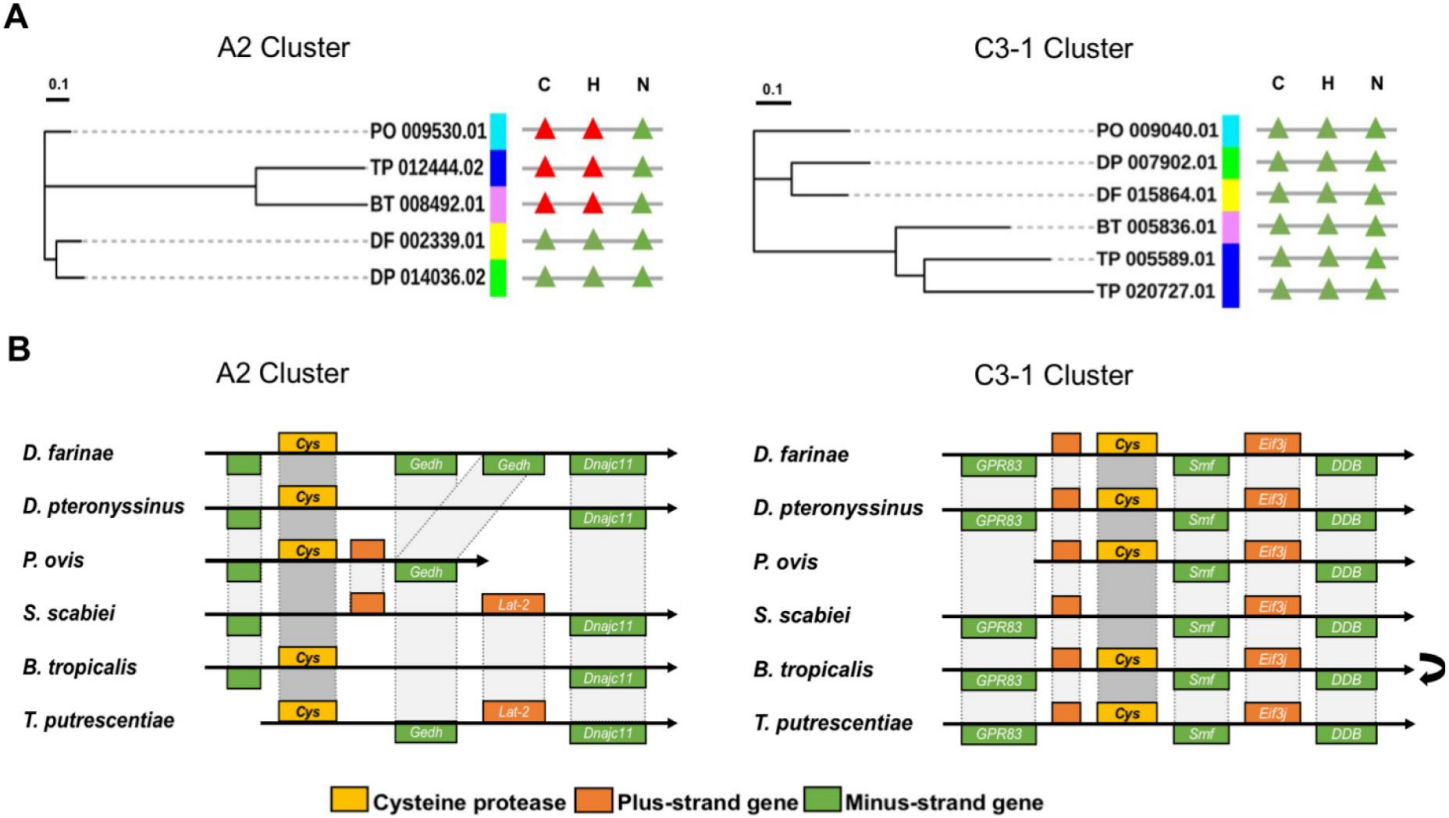
Cysteine protease homologs in the A2 and C3‐1 clusters. (A) Phylogenetic relationships and catalytic triads of cysteine proteases in the A2 and C3‐1 clusters were analyzed. Both clusters contained at least one cysteine protease from *Psoroptes ovis*, *Dermatophagoides pteronyssinus, Dermatophagoides farinae*, *Blomia tropicalis* and *Tyrophagus putrescentiae*, respectively. The active sites and site‐mutated sites of the catalytic triads (C‐H‐N) were marked in green triangles and red triangles, respectively. (B) Gene synteny alignment of cysteine proteases in the A2 cluster and C3‐1 cluster. For both A2 and C3‐1 cluster, no homolog was identified in *Sarcoptes scabiei*. The black arrow suggested after reverse complement.

Among the six astigmatic mite species, *S. scabiei* contained the smallest number of cysteine proteases and most of its T/PAGs were in the D1 cluster. In the C4 cluster, a total of 27 cysteine proteases were identified, whereas only one of them (SS_006399.01) was from *S. scabiei* (Supplementary Figure S5). Likewise, in the sub‐cluster C5‐2 that contained many T/PAGs of *D. farinae*, *D. pteronyssinus* and *P. ovis*, only one cysteine protease (SS_009273.01) was found in *S. scabiei* (Supplementary Figure S6). In the C2 cluster, we identified two conserved clusters A1 and A2, which contained closely related cysteine proteases from different astigmatic mites without tandem duplication (Figure 4A, Supplementary Figure S7). However, in the A2 cluster, there was no cysteine protease gene identified in *S. scabiei* (Figure 4A). Along with the A2 cluster, in the C3‐1 cluster, the active cysteine proteases can only be identified in the other five astigmatic mite species (Figure 4A). Selection pressure analysis identified 39 sites under negative selection in A2 cluster (Supplementary Table S4A). In the C3‐1 cluster, 5 sites were under positive selection and 44 sites were under negative selection (Supplementary Table S4B). The gene synteny alignment revealed that the putative genes of *S. scabiei* in both the A2 and C3‐1 clusters were absent, suggesting the gene decay of cysteine proteases in *S. scabiei* (Figure 4B, Supplementary Figure S8).

Allergenicity evaluation of cysteine proteases from *D. pteronyssinus* in *S. scabiei* gene decay clusters.

**FIGURE 5:**
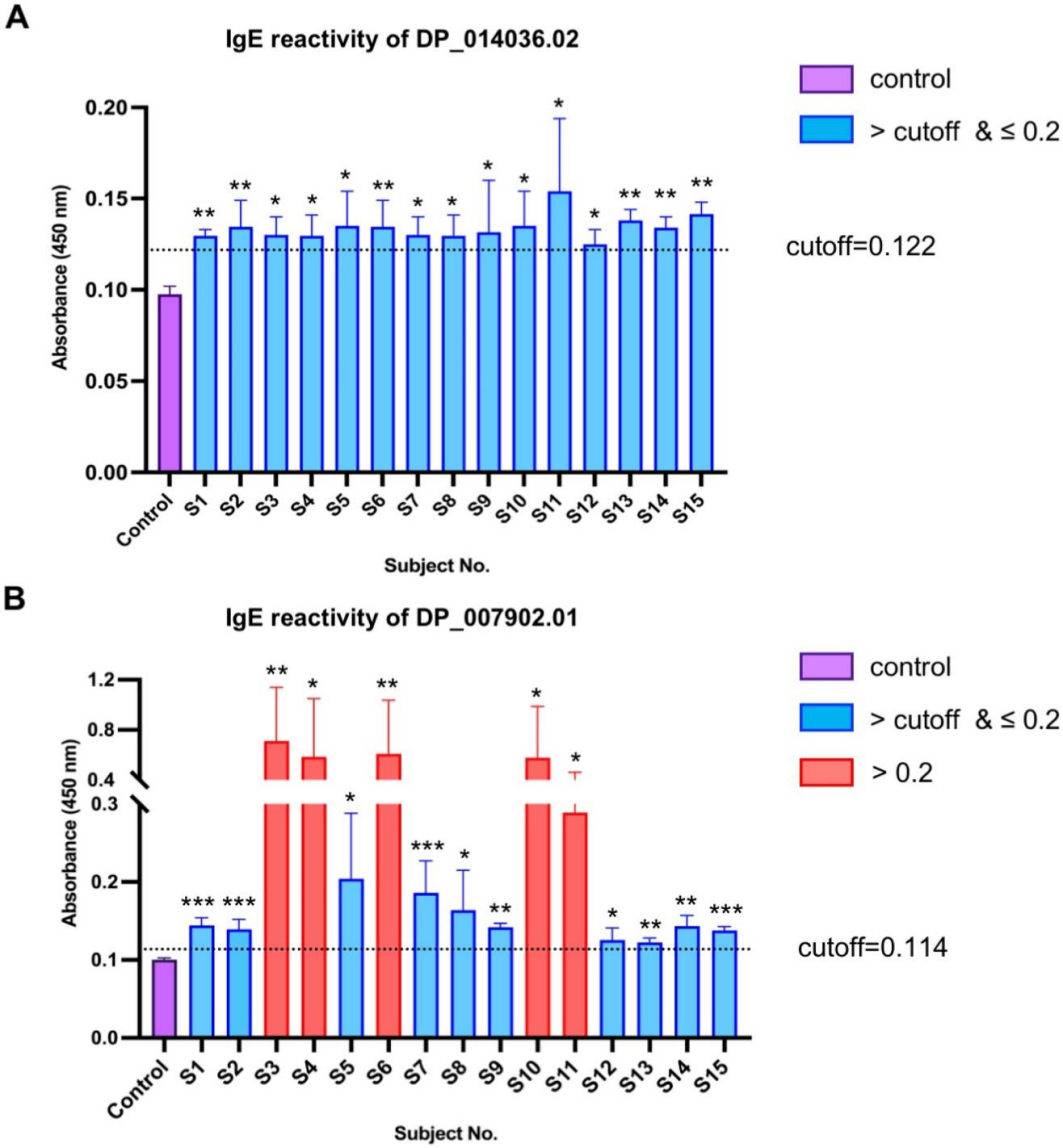
The IgE reactivity of cysteine proteases from *Dermatophagoides pteronyssinus* in the A2 and C3‐1 clusters. The allergenicity of cysteine protease (A) DP_014306.02 from A2 cluster and (B) DP_007902.01 from C3‐1 cluster was evaluated by IgE ELISA with sera of 15 HDM‐sensitized subjects (S1–S15) versus 8 non‐allergic subjects. Control represented the mean absorbance of 8 non‐allergic subjects. Data were shown as mean ± SEM. Cutoffs were calculated as the mean + 2SD of the non‐allergic subjects. Mean absorbance of HDM‐sensitized subjects greater than the cutoff but less than 0.2 was indicated in blue, and higher than 0.2 suggested in red. The statistical significance between each HDM‐sensitized subject and the non‐allergic subjects was determined by the Student's *t* test. ***p < 0.001, **p < 0.01, *p < 0.05. HDM, house dust mite.

The allergenicity of the cysteine proteases (DP_014036.02 and DP_007902.01) from *D. pteronyssinus* in the A2 and C3‐1 clusters was determined by IgE reactivity using ELISA with HDM‐sensitized patient sera. In comparison to the non‐allergic subjects, all of the 15 HDM‐sensitized subjects (100%) exhibited significantly elevated serum IgE levels in response to DP_014036.02 (Figure 5A). Likewise, the IgE sensitization rate of DP_007902.01 in patients achieved 100.0% as well, and 5 out of 15 HDM‐sensitized subjects (33.3%) had a high IgE binding (mean absorbance >0.2) (Figure 5B). Furthermore, the transcript expression level of DP_007902.01 was significantly higher than that of any other cysteine protease in *D. pteronyssinus*, except for Der p 1 (DP_002156.03) (Figure 3B). These findings demonstrated that both DP_014036.02 and DP_007902.01 had significant IgE reactivity. The exceptionally high IgE reactivity and significantly elevated transcript expression of DP_007902.01 suggested its potent allergenicity.

### 3.4 Integrated multi‐omics analysis of T/PAG clusters in storage mites

D2 cluster included 21 cysteine proteases of storage mite *B. tropicalis* and *T. putrescentiae*, with 5 genes from *B. tropicalis* and 16 genes from *T. putrescentiae*. All of the 5 genes from *B. tropicalis* and 5 out of 16 genes from *T. putrescentiae* were T/PAGs. The phylogenetic analysis was performed based on all the cysteine proteases in D2 cluster together with the reported Blo t 1 and Tyr p 1 (Supplementary Figure S9A). BT_013135.03 and BT_013137.01 were identified as two iso‐allergens of Blo t 1 and TP_010062.02 as Tyr p 1 (Supplementary Figure S9A and Table S1). All of the 5 genes from *B. tropicalis* and 8 of the total 16 genes from *T. putrescentiae* maintained the catalytic triads, indicating them as active cysteine proteases (Supplementary Figure S9A). Selection pressure analysis identified 3 sites under positive selection and 16 sites under negative selection in *B. tropicalis* (Supplementary Table S5A). For *T. putrescentiae*, 32 sites were positively selected, and 50 sites were negatively selected (Supplementary Table S5B). The transcript expression levels of BT_13135.03 and BT_13137.01 were significantly elevated compared to those of any other cysteine protease in the D2 cluster of *B. tropicalis* (Supplementary Figure S9B). In *T. putrescentiae*, the peptide sequences of IgE‐binding proteins in *T. putrescentiae*‐sensitized patient sera were identified by MALDI‐TOF MS previously^37^ and aligned to the cysteine protease genes. Interestingly, the best matched gene (TP_008599.01) was not in the D2 cluster and shared only 28.11% identity with the reported Tyr p 1 (Supplementary Figure S10 and Table S1). The transcript expression level of TP_008599.01 was significantly higher than that of any other cysteine protease from *T. putrescentiae* in D2 cluster (Supplementary Figure S9B).

Since *B. tropicalis* contained the largest number of T/PAGs among all the six astigmatic mites, C1‐1 and C5‐1, two tandem clusters of *B.* tropicalis were analyzed as well. For the 7 T/PAGs in the C1‐1 cluster, 6 of them were closely related, while only BT_001249.01 was distant (Supplementary Figure S11A). A total of 4 sites were under positive selection and 20 sites were under negative selection (Supplementary Table S6A). In addition, the catalytic triad analysis showed that BT_001249.01 was the only inactive gene in the C1‐1 cluster, with a mutation at the cysteine site (Supplementary Figure S11A). These findings suggested that all the T/PAGs except for BT_001249.01 in C1‐1 cluster were highly conserved and might be generated through recent gene duplication and expansion. As for C5‐1 cluster, it contained 21 cysteine protease genes with 19 of them as T/PAGs (Supplementary Figure S11B). The catalytic triad analysis identified 16 active genes while the 5 inactive genes showed site deletions (Supplementary Figure S11B). The selection pressure analysis showed that the C5‐1 cluster had 48 sites under positive selection and 131 sites under negative selection (Supplementary Table S6B).

## 4 DISCUSSION

Group 1 allergens are the major allergens in HDMs and are also identified in storage mites.^27^, ^38^, ^39^ They are characterized as papain‐like cysteine proteases and present in fecal pellets of HDMs, suggesting their function as digestive enzymes in mite intestinal tracts.^40^ In this study, we conducted a comparative genomic analysis of six astigmatic mites to reveal the evolutionary relationships of the cysteine protease family. Massive TAGs were identified, mainly in HDMs (*D. pteronyssinus*, *D. farinae*) and storage mites (*B. tropicalis* and *T. putrescentiae*) (Figure 1). This revealed that tandem duplication is the major feature of the rapid evolution of cysteine proteases in astigmatic mites. The cysteine protease DP_007902.01 was shown to have a significant IgE reactivity and a notable transcript expression level, which indicated it as a potential novel group 1 allergen of *D. pteronyssinus*. Gene decay events occurred in the skin‐burrowing parasitic mite *S. scabiei*, suggesting its adaptation to parasitism. Taken together, our comparative analysis provided insights into the evolution of cysteine proteases in astigmatic mites as well as the CRD of mite allergies.

In the D1 cluster, we identified Der p 1 (DP_002156.03), Der f 1 (DF_007090.03) and their closely related homologs (Figure 2). The sequence mapping of IgE‐binding proteins and the significantly high gene expression levels of DP_002156.03 and DF_007090.03 indicated their strong allergenicity (Figure 3, Supplementary Figure S1). Typically, proteins are considered to have cross‐reactivity with known allergens if they share a minimum of 35% sequence identity over an alignment of at least 80 amino acids.^41^ Meanwhile, the allergenicity of allergens has been suggested to be associated with their expression levels.^42^ Despite the low gene expression levels, all the cysteine protease homologs of *D. pteronyssinus* and *D. farinae* in D1 cluster shared more than 35% sequence identity with Der p 1 or Der f 1, and over half of them had >50% identity (Supplementary Table S3). To evaluate the allergenicity of cysteine proteases in the D1 cluster, the recombinant protein of DP_002156.02 was cloned, expressed and examined by ELISA with HDM‐sensitized patient sera, revealing an IgE sensitivity of 26.7% (Supplementary Figure S4). These results suggested that the cysteine protease homologs of HDMs in D1 cluster might have potential allergenicity and cross‐reactivity with Der p 1 and Der f 1.

We also examined the allergenicity of DP_014036.02 from the A2 cluster and DP_007902.01 from the C3‐1 cluster by ELISA with HDM‐sensitized patient sera. Although both DP_014036.02 and DP_007902.01 presented IgE reactivity of 100.0% (15/15), DP_007902.01 exhibited much higher allergenicity and transcript expression level (Figures 3B and 5). This suggested that DP_007902.01 might be a novel group 1 allergen of *D. pteronyssinus*. Epitope mapping identified multiple conserved and partially conserved sequences among cysteine proteases of A2, C3‐1 cluster and the group 1 allergens (Supplementary Figure S12). Interestingly, however, both DP_014036.02 and DP_007902.01 shared a sequence identity with Der p 1 (DP_002156.03) less than 35% (Supplementary Table S3). In the phylogenetic tree, C2 cluster consisted of the ancient cluster A1 and A2, the HDM cluster D1 and the storage mite cluster D2 (Figure 1). D1 and D2 cluster were closely related to A1 and A2 cluster, respectively. Therefore, we proposed that the cysteine proteases in the D1 cluster might have evolved from the A1 cluster, and the genes of the D2 cluster could have possibly originated from the A2 cluster. This might explain the low sequence identity between DP_014036.02 of the A2 cluster and Der p 1 (DP_002156.03) of the D1 cluster. Cysteine proteases from the C3 cluster were distant from the C2 cluster, suggesting a different lineage between DP_007902.01 and Der p 1 (DP_002156.03). Thus, although DP_007902.01 was demonstrated as a potential novel group 1 allergen that exhibited high allergenicity, the cross‐reactivity between DP_007902.01 and Der p 1 (DP_002156.03) still required further investigation.

Since animal parasites typically undergo genome reduction by gene loss over time,^43^ it was expected that the parasitic mite *P. ovis* and *S. scabiei* would exhibit a significantly reduced number of cysteine proteases. In this study, although *P. ovis* and *S. scabiei* harbored fewer cysteine proteases in comparison to the other four free‐living mite species, the difference in gene copy number between these two parasitic mite species and HDMs was marginal (Table 1). Hence, it is plausible that the astigmatic mite species typically possess a baseline number of cysteine proteases within the range of 20–29. Interestingly, gene decay events were observed exclusively in the skin‐burrowing parasitic mite *S. scabiei* (Figure 4, Supplementary Figure S8), suggesting a more obligatory parasitic lifestyle. As burrowing into the skin, *S. scabiei* has been reported to initially inhibit the inflammatory and immune response so as to escape from the host defense system and survive within the skin.^44^ Once *S. scabiei* can proliferate and establish their own population, the host inflammatory and allergic response will be induced. Previous studies suggested that the inactive protease paralogs in *S. scabiei* could protect them from complement mediated destruction by the host immune system.^45^, ^46^ Given this, we proposed that the putative cysteine proteases of *S. scabiei* in A2 and C3‐1 cluster might have the abilities to induce undesirable immune responses such as allergic reactions in the host. We examined the IgE reactivity of cysteine proteases from *D. pteronyssinus* in A2 and C3‐1 cluster, revealing their high allergenicity (Figure 5). Our findings suggested that the putative cystine proteases of *S. scabiei* in A2 and C3‐1 cluster may have initially exhibited significant allergenicity. The gene decay events of cysteine proteases in *S. scabiei* could possibly play an important role in preventing an overactive immune response, thereby contributing to the evasion of the host defense system.

Among the six astigmatic mite species, the storage mites *B. tropicalis* and *T. putrescentiae* had the largest number of cysteine proteases (Table 1). Massive tandem duplication events were also identified in *B. tropicalis* and *T. putrescentiae*, suggesting the rapid expansion of the cysteine protease family (Figure 1). Interestingly, *B. tropicalis* showed significantly higher expression of Blo t 1 (BT_13135.03 and BT_13137.01), while the reported Tyr p 1 (TP_010062.02) expressed at extremely low levels (Supplementary Figure S9B). Blo t 1 is not the major allergen of *B. tropicalis*, but it is still important because it has been reported to have 65% IgE reactivity in *B. tropicalis*‐sensitized patient sera.^27^ Unlike Der p 1 and Der f 1 that shared ∼81% sequence identity, Blo t 1 was reported to have only ∼35% sequence identity with Der p 1 and Der f 1^47^ ^,^ ^48^. This possibly explains the limited cross‐reactivity between Blot 1 and Der p 1.^49^ *T. putrescentiae* is a cosmopolitan mite commonly found in stored products and is considered as an important source of mite allergies.^50^ Unlike *D. pteronyssinus* and *D. farinae*, fewer allergens were reported in *T. putrescentiae*. In this study, the group 1 allergen identified by MS (TP_008599.01) exhibited a significantly higher expression than the reported Tyr p 1 (TP_010062.02). This indicated that the expression levels might play a critical role in the proteomic identification of group 1 allergens in *T. putrescentiae*.

## AUTHOR CONTRIBUTIONS

Ling Shi, Qing Xiong and Stephen Kwok Wing Tsui contributed to the conception and design of the study. Ling Shi, Qing Xiong, Fu Kiu Ao and Xiaojun Xiao were responsible for laboratory study. Ling Shi, Qing Xiong and Tsz Yau Wan performed data curation and bioinformatic analysis. Xiaoyu Liu, Baoqing Sun, Anchalee Tungtrongchitr assisted in data collection and interpretation of results. Ting Fan Leung provided clinical data. Ling Shi wrote the first draft of the manuscript. Ling Shi, Qing Xiong, Ting Fan Leung and Stephen Kwok Wing Tsui finalized the manuscript. All authors contributed to the manuscript and approved the final submitted version.

## CONFLICT OF INTEREST STATEMENT

We have no conflict of interest to disclose.

## Supporting information

Supplementary Material

## Data Availability

The genome and transcriptome sequencing data of the six astigmatic mites were available on the NCBI database (BioProject accession: PRJNA174061 for *D. farinae*, PRJNA388362 for *D. pteronyssinus*, PRJNA702011 for *B. tropicalis*, PRJNA706095 for *T. putrescentiae*, PRJNA521406 for *P. ovis*, PRJNA268368, PRJNA598457, PRJNA749654 and PRJNA304361 for *S. scabiei*).
