# Supplementary material for: Comparative analysis of cysteine proteases reveals gene family evolution of the group 1 allergens in astigmatic mites
Source: Clin Transl Allergy. 2023 Dec 15;13(12):e12324. doi: 10.1002/clt2.12324 (PMC10722327; doi:10.1002/clt2.12324)
Supplement: Supplementary file 1 — Supplementary Material [file CLT2-13-e12324-s001.pdf]

Supplementary materials

for

Comparative Analysis of Cysteine Proteases Reveals Gene Family Evolution of the Group 1 Allergens in Astigmatic Mites

Contents:

Supplementary Tables 1–8

Supplementary Figures 1–13

Supplementary Table 1 BLASTP search summary of identified group 1 allergens in six astigmatic mite genomes against the reported allergens

Species	Gene ID	Allergen	Accession	Identity (%)	Query cover (%)	E-value
D. pteronyssinus	DP_002156.03	Der p 1.0101	AAB60215.1	99.38	100	0.0
		Der p 1.0113	ABA39435.1	99.67	100	0.0
		Der p 1.0124	CAQ68250.1	99.67	100	0.0
D. farinae	DF_007090.03	Der f 1.0101	BAC53948.1	100	100	0.0
		Der f 1.0102	Q3HWZ4	99.64	100	0.0
		Der f 1.0108	ABL84749.1	99.69	100	0.0
		Der f 1.0109	ABL84750.1	99.69	100	0.0
		Der f 1.0110	ABL84751.1	99.69	100	0.0
B. tropicalis	BT_013135.03	Blo t 1.0101	AAK58415.1	99.69	100	0.0
	BT_013137.01	Blo t 1.0201	AAQ24541.1	98.80	100	$8x10^{-173}$
T. putrescentiae	TP_010062.02	Tyr p 1.0101	ABM53753.1	98.81	100	0.0
	TP_008599.01	Tyr p 1.0101	ABM53753.1	28.11	93	$1x10^{-41}$
P. ovis	PO_001230.04	Pso o 1	Q1EIQ30	99.68	100	0.0

Supplementary Table 2A Site-specific selection pressure analysis of cysteine protease genes from *D. pteronyssinus* in D1 cluster

Codon	d_S (α)	d_N (β)	d_N/d_S (ω)	Selection	P-value
33	0	2.153	NA	Positive	0.0464
38	0	0.649	NA	Positive	0.0404
172	0	13.417	NA	Positive	0.0399
221	0	19.343	NA	Positive	0.0229
284	0.08	17.694	221.175	Positive	0.0482
333	0	10.720	NA	Positive	0.0472
388	0	32.907	NA	Positive	0.0498
57	6.28	0.087	0.014	Negative	0.0123
62	2.475	0.162	0.06545	Negative	0.0272
85	8.949	0.575	0.06425	Negative	0.0479
88	1.981	0.124	0.06259	Negative	0.0084
89	10000	0.171	0.00002	Negative	0.0362
94	32.202	0.159	0.00494	Negative	0.0024
103	3.076	0.161	0.05234	Negative	0.0202
146	10000	0.179	0.00002	Negative	0.0008
157	1.513	0.129	0.08526	Negative	0.0254
184	2.318	0.083	0.03581	Negative	0.0133
185	2.391	0.3	0.12547	Negative	0.0425
188	2.471	0.076	0.03076	Negative	0.0072
189	1.853	0.244	0.13168	Negative	0.0412
193	1.43	0.156	0.10909	Negative	0.0366
230	2.9	0.109	0.03759	Negative	0.0157
261	10000	0.131	0.00001	Negative	0.0134
267	3.496	0.079	0.02260	Negative	0.0026
268	2.926	0.223	0.07621	Negative	0.0405
270	2.595	0.186	0.07168	Negative	0.019
297	1.131	0.077	0.06808	Negative	0.015
300	5.018	0.191	0.03806	Negative	0.0093
304	3.027	0.087	0.02874	Negative	0.0202
319	1.126	0.09	0.07993	Negative	0.0462
320	7.281	0.293	0.04024	Negative	0.0365
341	3.063	0.235	0.07672	Negative	0.0424
343	5.04	0.257	0.05099	Negative	0.0146
359	47.16	0.673	0.01427	Negative	0.0082
375	14.974	0.103	0.00688	Negative	0.0001
378	2.139	0.101	0.04722	Negative	0.0357

Supplementary Table 2B Site-specific selection pressure analysis of cysteine protease genes from *D. farinae* in D1 cluster

Codon	d_S (α)	d_N (β)	d_N/d_S (ω)	Selection	P-value
65	0	868.27	NA	Positive	0.0284
204	0	1.565	NA	Positive	0.0495
24	3677.041	0.24	0.00007	Negative	0.0379
31	4.247	0.104	0.02449	Negative	0.0468
42	2.003	0.076	0.03794	Negative	0.0251
54	1630.54	0.142	0.00009	Negative	0.0071
117	2.251	0.086	0.03821	Negative	0.0291
134	10000	0.132	0.00001	Negative	0.0315
146	4840.274	0.111	0.00002	Negative	0.0005
147	540.538	0.143	0.00026	Negative	0.0058
160	10000	0.08	0.00001	Negative	0.0022
163	768.941	0.141	0.00018	Negative	0.0045
181	7670.88	0.173	0.00002	Negative	0.0495
184	7.866	0.092	0.01170	Negative	0.0497
195	10000	0.089	0.00001	Negative	0.0029
230	8	0.122	0.01446	Negative	0.0191
237	10000	0.145	0.00001	Negative	0.0481
246	10000	0.111	0.00001	Negative	0.0047
261	10000	0.181	0.00002	Negative	0.0149
334	10000	0.176	0.00002	Negative	0.0423

Supplementary Table 2C Site-specific selection pressure analysis of cysteine protease genes from *S. scabiei* in D1 cluster

Codon	d_S (α)	d_N (β)	d_N/d_S (ω)	Selection	P-value
130	0	0.768	NA	Positive	0.045
172	0	2.266	NA	Positive	0.002
17	16.243	0.26	0.0160069	Negative	0.0372
19	20.977	0.353	0.01682795	Negative	0.0361
30	10000	0.297	0.0000297	Negative	0.0344
38	200.143	0.235	0.00117416	Negative	0.0107
41	10000	0.295	0.0000295	Negative	0.0341
46	10000	0.276	0.0000276	Negative	0.0052
47	10000	0.285	0.0000285	Negative	0.0383
66	9.868	0.071	0.00719497	Negative	0.0018
72	68.703	0.16	0.00232886	Negative	0.0248
78	39.824	0.649	0.01629671	Negative	0.0493
79	11.372	0.163	0.01433345	Negative	0.0014
80	6.293	0.191	0.03035118	Negative	0.0289
116	3.786	0.206	0.05441099	Negative	0.0096
118	5.124	0.286	0.05581577	Negative	0.0426
132	10000	0.362	0.0000362	Negative	0.007
134	1248.355	0.133	0.00010654	Negative	0.0059
151	1.028	0.138	0.13424125	Negative	0.0466
162	1787.3	0.145	8.1128E-05	Negative	0.0038
163	1.704	0.064	0.03755869	Negative	0.0061
166	3.02	0.068	0.02251656	Negative	0.0021
177	0.862	0.039	0.04524362	Negative	0.0121
184	2.347	0.124	0.0528334	Negative	0.0137
186	2.033	0.251	0.12346286	Negative	0.0384
184	2.347	0.124	0.0528334	Negative	0.0137
186	2.033	0.251	0.12346286	Negative	0.0384
220	1.759	0.071	0.04036384	Negative	0.0275
226	643.019	0.308	0.00047899	Negative	0.0488
229	0.893	0.034	0.03807391	Negative	0.0012
238	10000	0.179	0.0000179	Negative	0.0003
248	8.093	0.166	0.02051155	Negative	0.0058
256	15.608	0.244	0.01563301	Negative	0.0063
258	2.212	0.24	0.1084991	Negative	0.0451
260	4.541	0.317	0.06980841	Negative	0.0167
274	0.907	0.032	0.03528115	Negative	0.0013
276	1.362	0.029	0.02129222	Negative	0.001
277	2.723	0.171	0.06279838	Negative	0.0137
278	1.987	0.22	0.11071968	Negative	0.0241

279	1.736	0.215	0.12384793	Negative	0.0203
282	2.356	0.189	0.08022071	Negative	0.0393
294	0.862	0.159	0.18445476	Negative	0.0387
295	1.808	0.097	0.05365044	Negative	0.0282
299	746.053	0.116	0.00015548	Negative	0.0055
305	1.363	0.065	0.04768892	Negative	0.0059
306	10000	0.238	0.0000238	Negative	0.0267
330	9.081	0.261	0.02874133	Negative	0.0062
333	20.745	0.097	0.00467583	Negative	0.0161
335	21.866	0.119	0.00544224	Negative	0.0033
345	0.857	0.13	0.15169195	Negative	0.0476
349	1.646	0.089	0.05407047	Negative	0.0128
350	1.646	0.074	0.04495747	Negative	0.0054
353	1.085	0.034	0.03133641	Negative	0.0026
373	0.451	0.039	0.0864745	Negative	0.0456
374	2.405	0.037	0.01538462	Negative	0.0124
387	1.375	0.17	0.12363636	Negative	0.0434
388	0.903	0.044	0.04872647	Negative	0.0253
404	1.835	0.108	0.05885559	Negative	0.0089
405	1.834	0.171	0.09323882	Negative	0.0191

Supplementary Table 3 BLASTP search summary of cysteine proteases in D1, A2 and C3-1 cluster against Der p 1 and Der f 1

Species	Allergen ID	Homolog ID	Cluster	Identity (%)	Query cover (%)	E-value
D. pteronyssinus	DP_002156.03	DP_012865.03	D1	61.45	100	$2x10^{-145}$
		DP_015275.03	D1	60.78	100	$2x10^{-145}$
		DP_013414.03	D1	53.52	100	$3x10^{-129}$
		DP_015275.02	D1	51.24	96	$1x10^{-106}$
		DP_002156.02	D1	48.31	100	$1x10^{-107}$
		DP_012865.02	D1	46.08	98	$2x10^{-91}$
		DP_000828.01	D1	41.80	94	$2x10^{-83}$
		DP_013414.02	D1	39.94	97	$8x10^{-80}$
		DP_014036.02	A2	30.57	88	$6x10^{-42}$
		DP_007902.01	C3-1	30.45	48	$1x10^{-17}$
D. farinae	DF_007090.03	DF_005080.01	D1	57.83	96	$3x10^{-138}$
		DF_007090.04	D1	51.24	88	$4x10^{-73}$
		DF_005079.01	D1	43.88	100	$1x10^{-97}$
		DF_002339.01	A2	29.37	86	$9x10^{-35}$
		DF_015864.01	C3-1	30.82	80	$3x10^{-25}$

Supplementary Table 4A Site-specific selection pressure analysis of cysteine protease genes in A2 cluster

Codon	d_S (α)	d_N (β)	d_N/d_S (ω)	Selection	P-value
20	10000	0.206	0.000021	Negative	0.0108
61	28.553	0.062	0.002171	Negative	0.027
77	23.044	0.062	0.002691	Negative	0.0061
113	7.523	0.062	0.008241	Negative	0.0013
129	5.472	0.12	0.021930	Negative	0.0484
130	11.119	0.072	0.006475	Negative	0.0066
138	470.23	0.057	0.000121	Negative	0.0001
210	5.996	0.085	0.014176	Negative	0.0134
220	680.229	0.146	0.000215	Negative	<0.0001
227	1.6	0.073	0.045625	Negative	0.0193
233	4.469	0.056	0.012531	Negative	0.0073
252	24.927	0.259	0.010390	Negative	0.0246
253	9.803	0.041	0.004182	Negative	0.0031
255	10000	0.267	0.000027	Negative	0.0024
260	6.587	0.068	0.010323	Negative	0.0166
266	1.985	0.085	0.042821	Negative	0.0415
269	5.557	0.068	0.012237	Negative	0.0143
274	10000	0.113	0.000011	Negative	0.0045
278	10000	0.196	0.000020	Negative	0.0043
279	4.22	0.151	0.035782	Negative	0.0462
288	0.692	0.064	0.092486	Negative	0.0438
289	6786.13	0.058	0.000009	Negative	0.0002
292	10000	0.144	0.000014	Negative	0.004
300	10000	0.286	0.000029	Negative	0.0055
313	28.181	0.07	0.002484	Negative	0.0018
330	4.975	0.082	0.016482	Negative	0.0456
332	1.272	0.049	0.038522	Negative	0.0215
340	10000	0.104	0.000010	Negative	0.0004
342	6.876	0.209	0.030396	Negative	0.0459
348	6.547	0.065	0.009928	Negative	0.0182
370	680.229	0.146	0.000215	Negative	<0.0001
389	10000	0.14	0.000014	Negative	0.0001
398	1193.154	0.074	0.000062	Negative	0.0158
401	1.13	0.071	0.062832	Negative	0.0479
408	2.753	0.054	0.019615	Negative	0.0124
413	65.103	0.162	0.002488	Negative	0.0126
427	15.666	0.075	0.004787	Negative	0.0007
428	1.235	0.062	0.050202	Negative	0.0366
437	10000	0.446	0.000045	Negative	0.0402

Supplementary Table 4B Site-specific selection pressure analysis of cysteine protease genes in C3-1 cluster

Codon	d_S (α)	d_N (β)	d_N/d_S (ω)	Selection	P-value
15	0	42.149	NA	Positive	0.0298
283	0	2143.665	NA	Positive	0.0053
356	0	492.425	NA	Positive	0.0271
428	0	764.782	NA	Positive	0.0182
470	0	73.008	NA	Positive	0.0237
3	10000	0.15	0.000015	Negative	0.0001
13	24.266	0.215	0.008860	Negative	0.0424
48	10000	0.442	0.000044	Negative	0.0195
61	10000	0.598	0.000060	Negative	0.0227
68	1.61	0.094	0.058385	Negative	0.0261
69	3.254	0.101	0.031039	Negative	0.0251
71	292.333	0.071	0.000243	Negative	<0.0001
76	10000	0.062	0.000006	Negative	<0.0001
79	6.168	0.17	0.027562	Negative	0.0262
104	4287.338	0.067	0.000016	Negative	<0.0001
110	5010.109	0.28	0.000056	Negative	0.0063
113	2.443	0.073	0.029881	Negative	0.037
145	11.713	0.15	0.012806	Negative	0.0345
147	1.434	0.102	0.071130	Negative	0.0414
181	1185.153	0.223	0.000188	Negative	0.0026
182	6.445	0.212	0.032894	Negative	0.0384
191	292.333	0.236	0.000807	Negative	0.0409
192	1.565	0.105	0.067093	Negative	0.0483
203	4.52	0.062	0.013717	Negative	0.0067
204	8.184	0.148	0.018084	Negative	0.0221
210	10000	0.177	0.000018	Negative	0.0003
220	10.592	0.15	0.014162	Negative	0.01
221	10000	0.114	0.000011	Negative	0.0224
224	10000	0.474	0.000047	Negative	0.0255
234	10000	0.08	0.000008	Negative	<0.0001
257	7.09	0.391	0.055148	Negative	0.0336
261	10000	0.258	0.000026	Negative	0.0487
297	4576.117	0.081	0.000018	Negative	0.0265
305	5.429	0.182	0.033524	Negative	0.0352
314	1.392	0.144	0.103448	Negative	0.0498
317	21.404	0.06	0.002803	Negative	0.0151
365	3.58	0.208	0.058101	Negative	0.0476
385	10000	0.156	0.000016	Negative	0.0002
405	3.031	0.189	0.062356	Negative	0.0414
408	3.163	0.073	0.023079	Negative	0.0152

421	3660.026	0.182	0.000050	Negative	0.024
423	9	0.091	0.010111	Negative	0.0157
437	5.463	0.199	0.036427	Negative	0.0119
469	22.969	0.098	0.004267	Negative	0.0021
489	10.046	0.161	0.016026	Negative	0.012
491	5.182	0.218	0.042069	Negative	0.0173
517	343.169	0.082	0.000239	Negative	0.018
518	10000	0.194	0.000019	Negative	0.0178
519	2.319	0.178	0.076757	Negative	0.0372

Supplementary Table 5A Site-specific selection pressure analysis of cysteine protease genes from *B. tropicalis* in D2 cluster

Codon	d_S (α)	d_N (β)	d_N/d_S (ω)	Selection	P-value
75	0	94.353	NA	Positive	0.0182
133	0	2.367	NA	Positive	0.0438
314	0	12.656	NA	Positive	0.0303
12	14.438	0.132	0.009142541	Negative	0.0081
88	5605.881	0.259	4.62015E-05	Negative	0.0374
90	10000	0.664	0.0000664	Negative	0.0279
101	10.811	0.082	0.007584867	Negative	0.0429
117	10.922	0.083	0.007599341	Negative	0.0419
130	11.894	0.097	0.008155372	Negative	0.0289
158	10000	0.178	0.0000178	Negative	0.0399
194	8765.615	0.429	4.89412E-05	Negative	0.0468
198	10000	0.364	0.0000364	Negative	0.0106
205	1404.332	0.308	0.000219321	Negative	0.0487
221	130.488	0.122	0.000934952	Negative	0.0193
250	12.071	0.196	0.016237263	Negative	0.0356
303	663.206	0.097	0.000146259	Negative	0.0001
305	10.277	0.135	0.013136129	Negative	0.0245
312	4.301	0.082	0.019065334	Negative	0.0103
338	9.492	0.111	0.011694058	Negative	0.0306

Supplementary Table 5B Site-specific selection pressure analysis of cysteine protease genes from *T. putrescentiae* in D2 cluster

Codon	d_S (α)	d_N (β)	d_N/d_S (ω)	Selection	P-value
7	0.186	6.051	32.532	Positive	0.0167
20	0.241	101.441	420.917	Positive	0.0387
23	0.455	94.7	208.132	Positive	0.0063
26	1.238	10000	8077.544	Positive	0.0062
29	0	534.209	NA	Positive	0.0088
96	0.038	12.377	325.711	Positive	0.0061
101	0.936	26.486	28.297	Positive	0.0249
112	0.378	23.811	62.992	Positive	0.0042
116	0.7	131.996	188.566	Positive	0.0174
126	0	3.489	NA	Positive	0.0327
160	0	9.491	NA	Positive	0.0361
173	0.168	3.854	22.940	Positive	0.0487
192	0.201	29.607	147.299	Positive	0.0233
216	0	1.95	NA	Positive	0.0471
229	0	2.613	NA	Positive	0.0371
232	0	26.827	NA	Positive	0.0133
233	0	15.757	NA	Positive	0.0186
247	0.304	14.01	46.086	Positive	0.0271
261	0.592	73.813	124.684	Positive	0.0337
279	0.041	43.5	1060.976	Positive	0.0105
281	0.208	305.093	1466.793	Positive	0.0182
282	0.299	149.53	500.100	Positive	0.0374
283	0.053	9.208	173.736	Positive	0.0015
287	0.48	20.916	43.575	Positive	0.0123
288	0.126	38.507	305.611	Positive	0.0157
300	0.547	403.072	736.878	Positive	0.0057
314	0	2.061	NA	Positive	0.0331
316	0	5.219	NA	Positive	0.0005
327	0	53.159	NA	Positive	0.0203
398	0.513	252.901	492.984	Positive	0.0289
400	0	8.569	NA	Positive	0.0199
404	0	6.13	NA	Positive	0.0357
5	10000	0.245	0.0000245	Negative	0.0169
21	2.231	0.317	0.14208875	Negative	0.0111
39	6.745	0.234	0.03469236	Negative	0.0448
52	1983.846	0.169	8.5188E-05	Negative	0.0029
56	6.512	0.342	0.05251843	Negative	0.0121
60	10000	0.618	0.0000618	Negative	0.0219
63	10000	0.426	0.0000426	Negative	0.0003
92	94.164	0.447	0.00474704	Negative	0.0361

93	58.316	0.489	0.00838535	Negative	0.0013
97	93.71	0.527	0.00562373	Negative	0.0025
99	8.076	0.615	0.07615156	Negative	0.024
100	3.585	0.361	0.10069735	Negative	0.0104
103	2.502	0.308	0.12310152	Negative	0.0093
168	6.985	0.401	0.05740873	Negative	0.0048
171	20.946	0.802	0.03828893	Negative	0.0025
172	4.38	0.172	0.03926941	Negative	0.0089
175	0.885	0.086	0.09717514	Negative	0.0211
180	1.168	0.136	0.11643836	Negative	0.0045
184	1.446	0.291	0.20124481	Negative	0.035
188	5.162	0.62	0.12010849	Negative	0.0204
194	5.855	0.771	0.13168232	Negative	0.0246
198	0.54	0.056	0.1037037	Negative	0.0314
199	10000	0.444	0.0000444	Negative	0.0171
201	4.094	0.365	0.08915486	Negative	0.0076
202	19.639	0.159	0.00809614	Negative	<0.0001
203	8.311	0.114	0.01371676	Negative	0.0017
204	1.586	0.082	0.0517024	Negative	0.0067
205	0.584	0.059	0.1010274	Negative	0.0211
224	1.357	0.266	0.19602063	Negative	0.0329
231	1.612	0.223	0.13833747	Negative	0.0187
234	1.809	0.248	0.13709232	Negative	0.0119
239	1.308	0.15	0.1146789	Negative	0.0086
240	0.267	1.564	5.8576779	Negative	0.0481
245	2.426	0.051	0.02102226	Negative	0.0005
260	4.778	0.368	0.07701967	Negative	0.0268
293	11.65	1.021	0.08763948	Negative	0.0425
298	1.52	0.126	0.08289474	Negative	0.0031
309	0.531	0.044	0.08286252	Negative	0.0143
315	0.794	0.045	0.05667506	Negative	0.0071
322	7.588	0.086	0.01133368	Negative	0.0001
328	6.777	0.35	0.05164527	Negative	0.0145
330	2.788	0.28	0.10043042	Negative	0.0079
331	7.411	0.342	0.04614762	Negative	0.0496
348	1.084	0.161	0.14852399	Negative	0.0174
356	1.708	0.216	0.1264637	Negative	0.0046
363	0.602	0.051	0.08471761	Negative	0.0495
366	0.864	0.087	0.10069444	Negative	0.0167
367	1.553	0.043	0.02768835	Negative	0.0001
379	2.099	0.225	0.1071939	Negative	0.0132
393	2.237	0.261	0.11667412	Negative	0.0208

Supplementary Table 6A Site-specific selection pressure analysis of cysteine protease genes from *B. tropicalis* in C1-1 cluster

Codon	d_S (α)	d_N (β)	d_N/d_S (ω)	Selection	P-value
27	0.000	1.539	NA	Positive	0.0448
193	0.000	62.744	NA	Positive	0.0146
248	0.000	3.311	NA	Positive	0.0246
286	0.537	16.324	30.39851	Positive	0.0115
29	872.777	0.508	0.00058	Negative	0.005
43	16.339	0.394	0.02411	Negative	0.0104
45	292.333	0.436	0.00149	Negative	0.0109
46	26.452	0.164	0.00620	Negative	0.0169
48	5.127	0.178	0.03472	Negative	0.0109
52	2.025	0.181	0.08938	Negative	0.0373
53	4.869	0.2	0.04108	Negative	0.0394
63	61.656	0.202	0.00328	Negative	0.0176
119	2.846	0.242	0.08503	Negative	0.0446
122	1713.786	0.242	0.00014	Negative	0.0006
126	11.677	0.402	0.03443	Negative	0.07
155	8.185	0.248	0.03030	Negative	0.0256
162	4.549	0.254	0.05584	Negative	0.0257
182	821.225	0.384	0.00047	Negative	0.0109
190	65.743	0.193	0.00294	Negative	0.0042
216	10.000	0.236	0.02360	Negative	0.0135
230	10000.000	0.161	0.00002	Negative	0.0001
239	3.381	0.177	0.05235	Negative	0.0223
243	2.607	0.221	0.08477	Negative	0.0443
249	7.920	0.671	0.08472	Negative	0.0384

Supplementary Table 6B Site-specific selection pressure analysis of cysteine protease genes from *B. tropicalis* in C5-1 cluster

Codon	d_S (α)	d_N (β)	d_N/d_S (ω)	Selection	P-value
8	0	8.455	NA	Positive	0.0042
13	0.328	70.041	213.5396	Positive	0.0175
15	0.239	80.077	335.0502	Positive	0.0021
39	0.71	478.565	674.0352	Positive	0.0052
118	0.207	23.385	112.9710	Positive	0.0008
141	0	3.596	NA	Positive	0.0416
156	0.864	58.003	67.1331	Positive	0.0209
176	0.207	149.289	721.2029	Positive	0.0279
219	0.596	13.215	22.1728	Positive	0.0226
221	0	53.854	NA	Positive	0.0004
226	0.586	120.65	205.8874	Positive	0.0005
251	0	29.55	NA	Positive	0.0017
253	0	7.124	NA	Positive	0.0229
269	0	3.977	NA	Positive	0.0316
273	0	20.956	NA	Positive	0.0219
285	0	2.402	NA	Positive	0.0284
305	0.221	51.442	232.7692	Positive	0.0015
313	0	6.151	NA	Positive	0.0423
314	0.384	30.297	78.8984	Positive	0.0231
320	0.059	49.497	838.9322	Positive	0.018
344	0	10.3	NA	Positive	0.0122
417	0.512	19.285	37.6660	Positive	0.0422
422	0.414	5.032	12.1546	Positive	0.0284
425	0.654	301.848	461.5413	Positive	0.0342
429	0.157	95.459	608.0191	Positive	0.0438
430	0.198	286.29	1445.9091	Positive	0.0001
2	2.4	0.184	0.0767	Negative	0.0379
4	2.796	0.14	0.0501	Negative	0.005
5	4.639	0.425	0.0916	Negative	0.0036
12	8.612	0.79	0.0917	Negative	0.0112
17	1.833	0.297	0.1620	Negative	0.0194
32	2.35	0.127	0.0540	Negative	0.0089
38	6.446	0.434	0.0673	Negative	0.0219
78	0.586	0.074	0.1263	Negative	0.0355
79	2.666	0.156	0.0585	Negative	0.0181
87	3.866	0.071	0.0184	Negative	0.0004
93	1.125	0.092	0.0818	Negative	0.0213
99	0.619	0.076	0.1228	Negative	0.0499
107	3.251	0.182	0.0560	Negative	0.0028
113	9.089	0.191	0.0210	Negative	0.002

116	5.891	0.083	0.0141	Negative	0.0014
123	1.047	0.136	0.1299	Negative	0.0467
124	1.663	0.234	0.1407	Negative	0.0169
144	2.052	0.094	0.0458	Negative	0.0025
148	1.801	0.258	0.1433	Negative	0.0391
157	1.87	0.071	0.0380	Negative	0.0014
162	24.199	0.928	0.0383	Negative	0.011
167	13.632	0.664	0.0487	Negative	0.0432
169	5.121	0.545	0.1064	Negative	0.0377
172	6.851	0.307	0.0448	Negative	0.0336
179	13.384	0.819	0.0612	Negative	0.0429
180	197.607	1.136	0.0057	Negative	0.0145
183	10000	0.75	0.0001	Negative	0.0439
225	3.573	0.391	0.1094	Negative	0.0139
229	2.511	0.152	0.0605	Negative	0.0282
250	10000	0.761	0.0001	Negative	0.0009
255	6.742	0.333	0.0494	Negative	0.0013
257	3.135	0.081	0.0258	Negative	0.0102
259	4.722	0.512	0.1084	Negative	0.0099
265	1.231	0.071	0.0577	Negative	0.0018
268	2.189	0.228	0.1042	Negative	0.0095
274	0.963	0.086	0.0893	Negative	0.0247
276	2.904	0.168	0.0579	Negative	0.0009
279	1.386	0.187	0.1349	Negative	0.0401
281	3.315	0.173	0.0522	Negative	0.0214
306	1.893	0.076	0.0401	Negative	0.0136
307	15.446	0.722	0.0467	Negative	0.0143
327	2.833	0.178	0.0628	Negative	0.0301
329	1.23	0.137	0.1114	Negative	0.0094
330	1.208	0.06	0.0497	Negative	0.0038
341	2.425	0.104	0.0429	Negative	0.0185
347	1.451	0.141	0.0972	Negative	0.0037
356	1.624	0.122	0.0751	Negative	0.0128
360	3.372	0.458	0.1358	Negative	0.0391
366	4.977	0.498	0.1001	Negative	0.0483
376	2.088	0.071	0.0340	Negative	0.009
380	7.754	0.643	0.0829	Negative	0.0243
383	2.916	0.121	0.0415	Negative	0.0006
384	2.619	0.256	0.0977	Negative	0.0155
389	4.373	0.234	0.0535	Negative	0.0011
390	1.966	0.267	0.1358	Negative	0.0312
406	1.979	0.226	0.1142	Negative	0.0213
425	0.921	0.075	0.0814	Negative	0.0129

426	2.48	0.106	0.0427	Negative	0.0029
427	1.028	0.089	0.0866	Negative	0.0167
428	4.915	0.105	0.0214	Negative	0.0019
431	2.409	0.286	0.1187	Negative	0.0212
437	18.007	0.456	0.0253	Negative	0.0405
438	13.18	0.976	0.0741	Negative	0.0055
440	3.823	0.396	0.1036	Negative	0.0443
442	5.221	0.837	0.1603	Negative	0.0313
448	9871.428	0.235	0.0000	Negative	0.0003
450	2.255	0.506	0.2244	Negative	0.0706
452	10000	0.374	<0.0001	Negative	0.0164

Supplementary Table 7 The information of serum samples for ELISA

Sample ID	Subject No.	Gender	Age (years)	RAST to Der p 1 (Class)	Der p 1-specific IgE (kUA/l)
BC62	Control	Female	7.2	0	<0.35
BC79	Control	Male	5.9	0	<0.35
BC80	Control	Male	6.9	0	<0.35
BC127	Control	Female	13.2	0	<0.35
BC129	Control	Female	13.1	0	<0.35
BC149	Control	Male	8.7	0	<0.35
BC231	Control	Female	14.8	0	<0.35
BC237	Control	Female	14.9	0	<0.35
BC126	S1	Female	13.2	5	79.9
BC130	S2	Female	13.3	6	>100
BC133	S3	Male	13.3	6	>100
BC160	S4	Female	13.0	5	52.3
BC162	S5	Female	14.0	5	66.9
BC169	S6	Female	13.3	5	94.7
BC177	S7	Male	13.0	5	97.8
BC182	S8	Female	10.5	6	>100
BC194	S9	Male	11.0	6	>100
A751	S10	Female	14.0	5	87.7
A765	S11	Male	9.8	5	74.3
BC78	S12	Male	6.1	4	39.4
BC105	S13	Male	17.5	4	39.3
BC146	S14	Female	13.1	4	19.7
BC190	S15	Male	12.5	4	40.7

* RAST class to Der p 1 of each subject was measured by a fluorescent enzyme immunoassay based on ImmunoCAP technology (AutoCAP system, Phadia AB, Uppsala, Sweden).

Supplementary Table 8 The protein sequences of cysteine proteases

Gene ID	Protein sequences
DP_002156.02	MKFEIIFTIFLIVTIKTHEPIKTFEQFKKVFHKQYSNINEENTRRKN<br>FENALKYVEGNKRRGGVKITQFADLSLEEFKSRYIMDAKTYANV<br>KKRNFYSSGKCQIKDIHLPASIDLRKSGHVTPIQNQGLCGSCWAF<br>SAIAAAESAFLASNISVNLSVQELVDCASDHGCNGDDTITGFDYI<br>MENGVVIENVYPYTGYKNNCNRHDTKRYKILSYCLISSQDENKI<br>KQTLAITKSAISVVITIGDIDEFRYYDGNTLIRYDKNYGSNYHAV<br>NIVGYGTIQGVDYWIVRNSWGEYWGADGYAYLEMHKDLMGID<br>EYPLVAIV
DP_007902.01	MMKSSLIILFVSIICCWQQILADTPANCTYEDIKGLWLFEETEPIK<br>DRWEKCPEHQQQREKYSKKIFIRLDFPNVAVDKFGNIGEWTMIY<br>NQGFEVKINYRKYFAFSAYERKSENNVLSYCHKTQPGWSHDVL<br>GNNWACYVGHKVNNWNDDVSKTVSAEKFPVKQHSERDLYLQ<br>NINVEHILSQKHIDHLNSQQKSWKAIVYPDLQTKSIEHLIQMAGG<br>RKSRIISRPKPLRATEQQKQLARSLPESFDWRNLNGIDYVSPVRD<br>QGKCGSCYTFASMAMLESRIRIQTNNTFKPIFSTQEVVDCSEYSQ<br>GCDGGFSYLIAGKYAQDFGVIDESCYPYKGVTGKCQNQQNFNQ<br>TNEKCKQRTYTIDYKYVGGYFGACNEEAMQIELVQNGPIAVGFE<br>VYGDFFGYSKGIYSHQPSNESNDQHKPTIKAEFNPFEMTNHAVLI<br>VGYGKDKKTGEKYWIVKNSWGKQWGMDGYFWMRRGTDECA<br>IESLAMAATPIPN
DP_014036.02	MIPYSILSTLLSLIILDYQQSAAMEQLHQQLTIADSNKNIPAQSAQ<br>VEYLQFIRSTYGRQNLTEIAEEFDLKNFEQNFTEFQKKYDKHYF<br>DGEDLEIRRANFLYSLKVVKDNDNDYKTGRVPYRLKINKFADFG<br>MNEKIFINQKLLTAKHETANKPIPFNNNHSKISMNVVRKMNQTSI<br>TFPAQFDWRSQSMVTTIEDQGQCGSSWIFAGVGLVESANSIAGN<br>PLVVLSKQQVLDCVLAPFYLSDGCNGGMLDDVFHYARQVGLT<br>MEKTYPYKGKQGECRQVLGEWREWIYRYNYLPLNSSDEMIMW<br>VVYNKGPVATLINAGDRHFQLYSGGILTDGGDRGLSGEHDQYV<br>QIIGWGNDNGLDFWLIKNSWGRDWGESGYARMIRGVNNRGINT<br>VVAYAEALPIRQPEPPKPTTEKPEPDASIGMFEHSNQMTIMMITLI<br>FSIVILILFN

D. farinae

```
DF_007090.03 108 ACRINS-VNVPSELDLRSLRTVT 111   174 TIPRGIEYIQQNGVVEERSYPYVAREQQCRR 212
DF_005079.01 100 DCELHGDFNPPKEFDLRP--HLT 129   182 TVLDGIEYIMANGTTTEEACPFISEESTCDQ 204
DF_007090.04 100 GCEITQ-KNVPASLDLLSMGYVT 122   174 APPTAFKYLQKTGVVDAKVYPYFPRRAMSLT 204
DF_005080.01 100 FCEITA-RTTIAALDLRSLNHLT 122   174 TVPRGLDYIQENGIVEEQVYEYNARENYCEP 204
```

D. pteronyssinus

```
DP_002156.03 173 TIPRG-------IEYIQHNGVVQESYYRYVAREQ 199   200 SCRRPNA--QRFGISNY 215
DP_013414.03 176 TIPRG-------LDYIQQNGIVEEQAYEYNAREN 202   203 NCEPPEN--PRHSIEQY 218
DP_002156.02 173 DTITG-------FDYIMENGVVIENVYPYTGYKN 199   200 NCNRHDT--KRYKILSY 215
DP_015275.03 183 TIDQG-------LYYIQRNGILSEHRYPYVAREQ 209   207 SCQRPSLNSRGYSIQNY 227
DP_012865.03 180 NIEQG-------LYYIQHSGIVQENLYPYVEREQ 206   210 SCQRPSLNSRGYPIQNY 224
DP_015275.02 172 NIQRGLNDLKFG---------------------- 183   187 ----------RYVTYSY 191
DP_012865.02 139 NIQRGLNDLKLGIVYIDNYGVVKESDYPYVEHAQ 173   173 LCRRLQG-VSRYKTDNY 189
DP_000828.01 214 YIFLA-------LDYVLKHGVLTDKSYPYERITQ 240   241 KCELPKI-GKRYHIKNW 257
DP_013414.02 189 KTAIA-------FNYLVTNGTTTQKAYPYTAKEG 215   216 ACDPPEE--PRYILENW 231
```

Supplementary Figure 1 The alignment of cysteine proteases from HDMs in D1 cluster

The peptide sequences of IgE-binding proteins in HDM-sensitized patient sera detected by mass spectrometry (highlighted in yellow) were aligned to the cysteine proteases. Der f 1 (DF_007090.03) and Der p 1 (DP_002156.03) were marked as red in label.

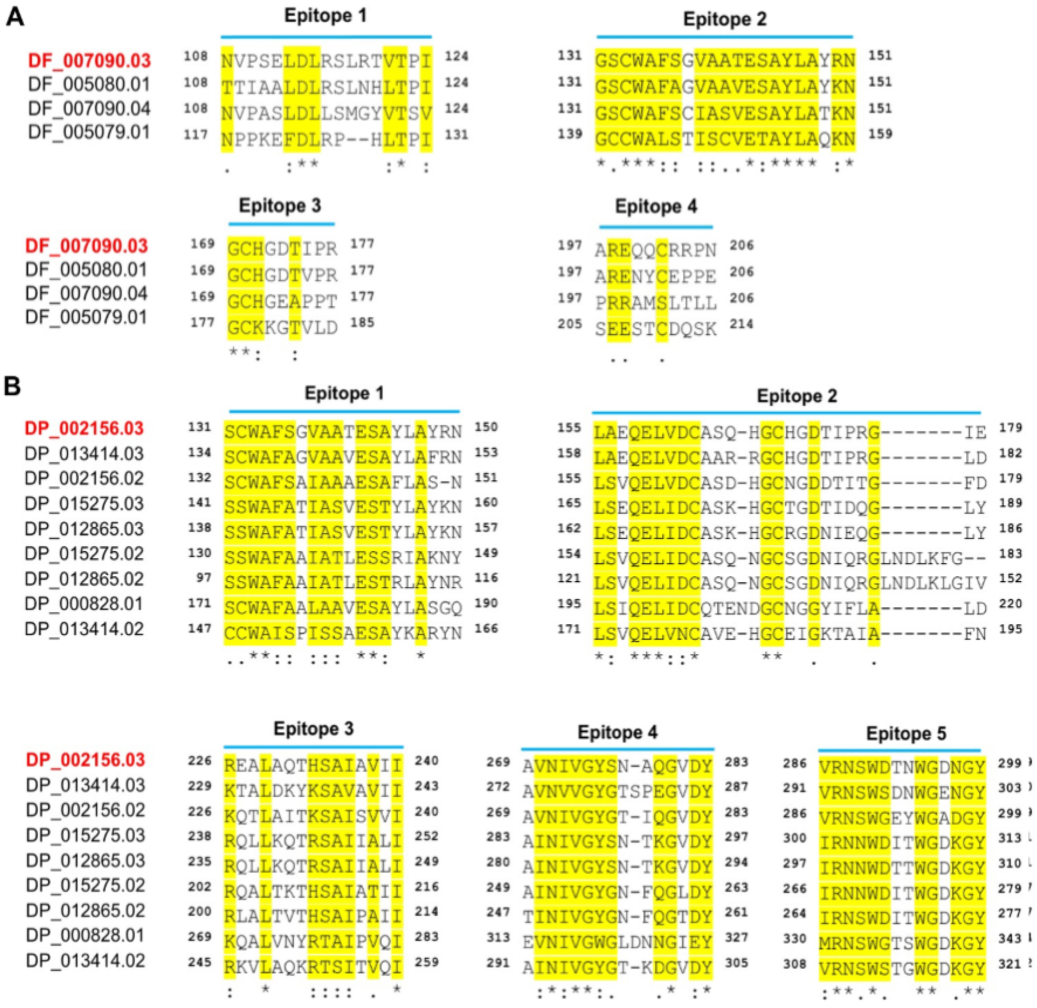

Supplementary Figure 2 The epitope mapping of cysteine proteases from HDMs in D1 cluster

The linear IgE epitope sequences of Der f 1 and Der p 1 were retrieved from The Immune Epitope Database (IEDB) and aligned to the cysteine proteases of **(A)** *D. farinae* and **(B)** *D. pteronyssinus* in D1 cluster. Der f 1 (DF_007090.03) and Der p 1 (DP_002156.03) were marked in red. The conserved site was denoted as " * ", conservative mutation as " : ", semi-conservative mutation as " . ". The sequences that are conserved, conservative mutated or semi-conservative mutated were highlighted in yellow.

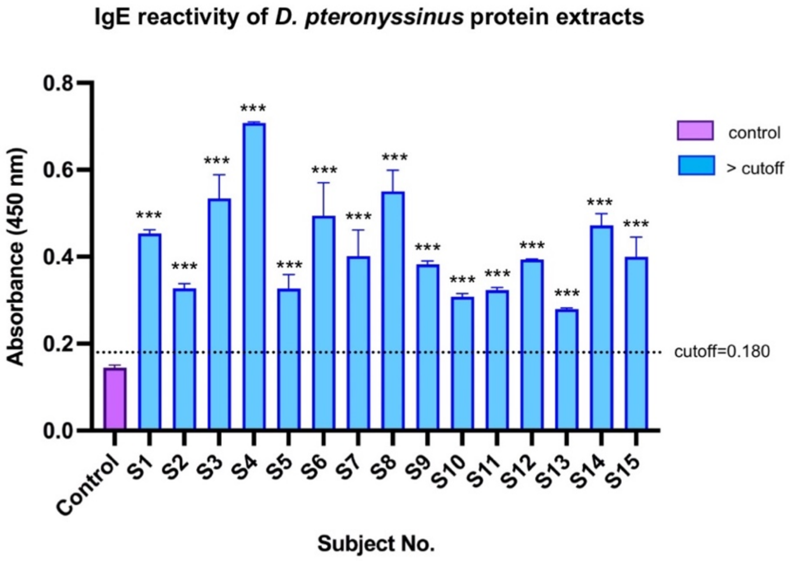

Supplementary Figure 3 The IgE reactivity of *D. pteronyssinus* protein extracts

The allergenicity of *D. pteronyssinus* extracts which contained the natural Der p 1 was evaluated by IgE ELISA with sera of 15 HDM-sensitized subjects (S1-S15) versus 8 non-allergic subjects. Control represented the mean absorbance of 8 non-allergic subjects. Data were shown as mean ± SEM. Cutoffs were calculated as the mean + 2SD of the non-allergic subjects. The statistical significance between each HDM-sensitized subject and the non-allergic subjects was determined by Student's t test. *** $P < 0.001$.

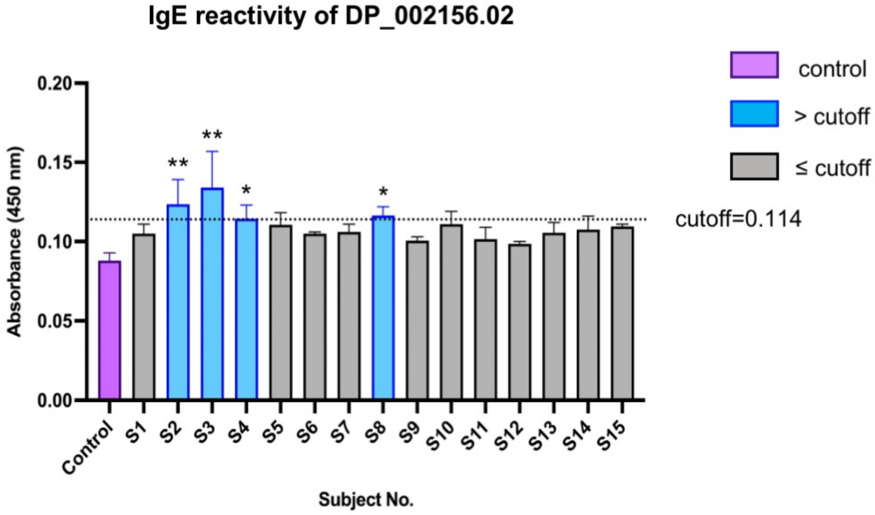

Supplementary Figure 4 The IgE reactivity of the cysteine protease from HDMs in D1 cluster

The allergenicity of DP_002156.02, the tandemly arrayed homolog of Der p 1 (DP_002156.03) was evaluated by IgE ELISA with sera of 15 HDM-sensitized subjects (S1-S15) versus 8 non-allergic subjects. Control represented the mean absorbance of 8 non-allergic subjects. Data were shown as mean ± SEM. Cutoffs were calculated as the mean + 2SD of the non-allergic subjects. The statistical significance between each HDM-sensitized subject and the non-allergic subjects was determined by Student's t test. ** $P < 0.01$, * $P < 0.05$.

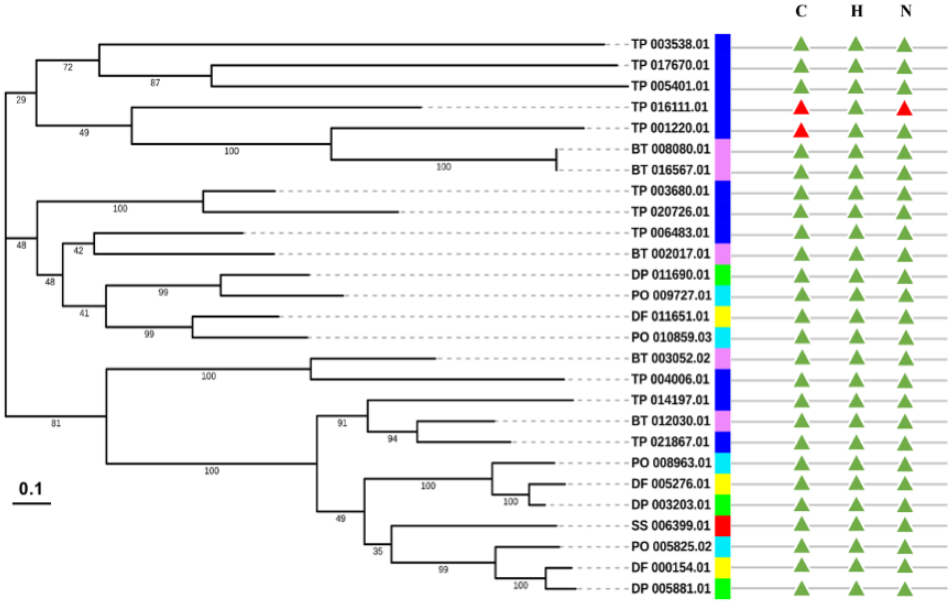

Supplementary Figure 5 Phylogenetic analysis and catalytic triad alignment of cysteine proteases in C4 cluster

C4 cluster contained 27 cysteine proteases, 11 from *T. putrescentiae*, 5 from *B. tropicalis*, 3 from *D. farinae*, 3 from *D. pteronyssinus*, 4 from *P. ovis* and 1 from *S. scabiei*. No T/PAG was identified in this cluster. The catalytic triads (C-H-N) were aligned, with the active sites and site-mutated sites marked in green triangles and red triangles, respectively.

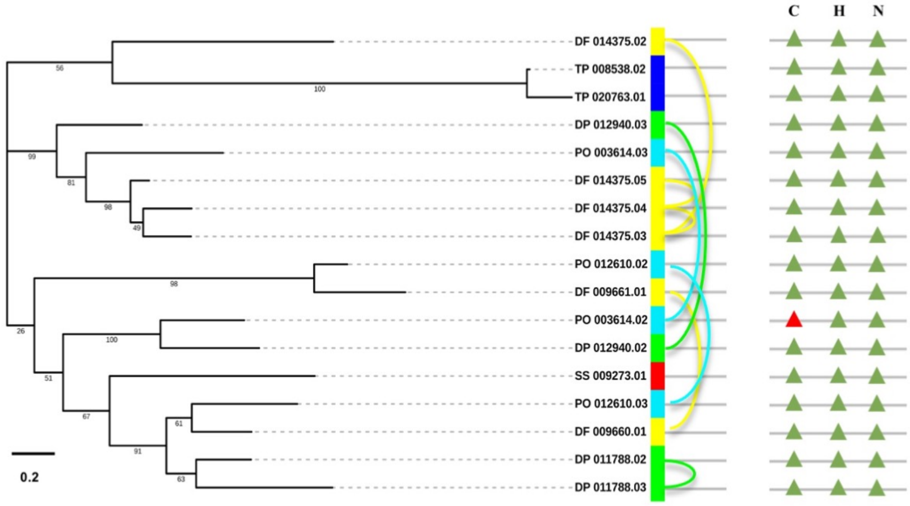

Supplementary Figure 6 Phylogenetic analysis and catalytic triad alignment of cysteine proteases in C5-2 cluster.

C5-2 cluster contained 17 cysteine proteases, 6 from *D. farinae*, 4 from *D. pteronyssinus*, 2 from *T. putrescentiae*, 4 from *P. ovis* and 1 from *S. scabiei*. The TAGs were connected by solid curved lines. The active sites and mutated sites of the catalytic triads (C-H-N) were marked in green triangles and red triangles, respectively.

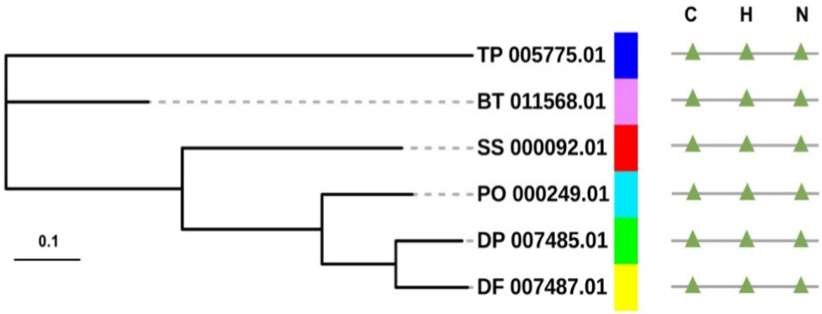

Supplementary Figure 7 Phylogenetic analysis and catalytic triad alignment of cysteine proteases in A1 cluster

A1 cluster contained 6 active cystine protease genes from *D. farinae*, *D. pteronyssinus*, *B. tropicalis, T. putrescentiae*, *P. ovis* and *S. scabiei* respectively. This is a conserved cluster that no T/PAG was identified. The active sites of the catalytic triads (C-H-N) were marked in green triangles.

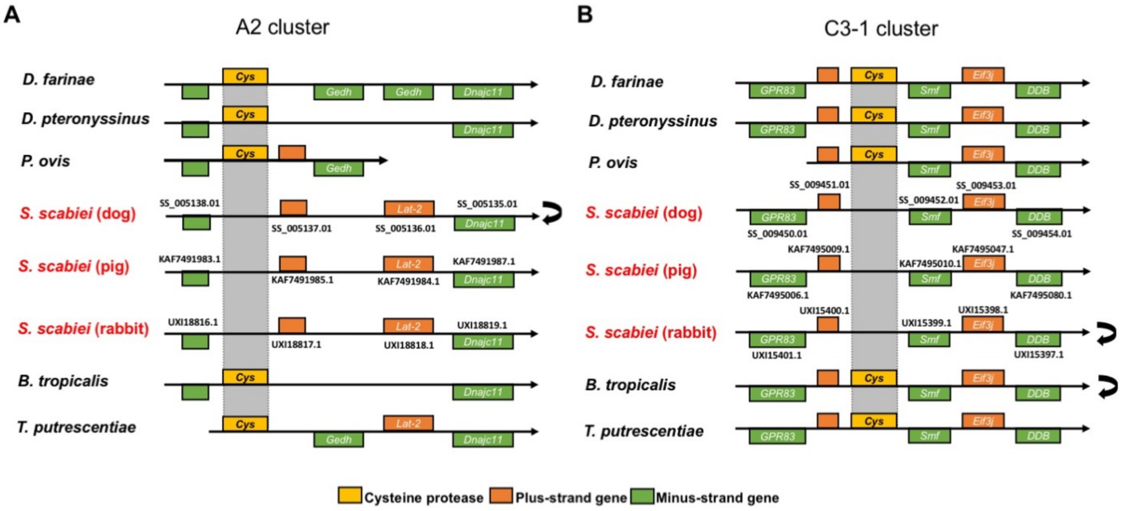

Supplementary Figure 8 The gene synteny alignment of cysteine proteases in A2 and C3-1 cluster with three different *S. scabiei* genomes

The gene synteny of cysteine proteases in **(A)** A2 cluster and **(B)** C3-1 cluster was examined with *S. scabiei* genomes from dog (BioProject accession: PRJNA268368), wild pig (BioProject accession: PRJNA598457) and European rabbit (BioProject accession: PRJNA749654). The gene IDs in three *S. scabiei* genomes were denoted as well. No cysteine protease was identified in any of the three *S. scabiei* genomes.

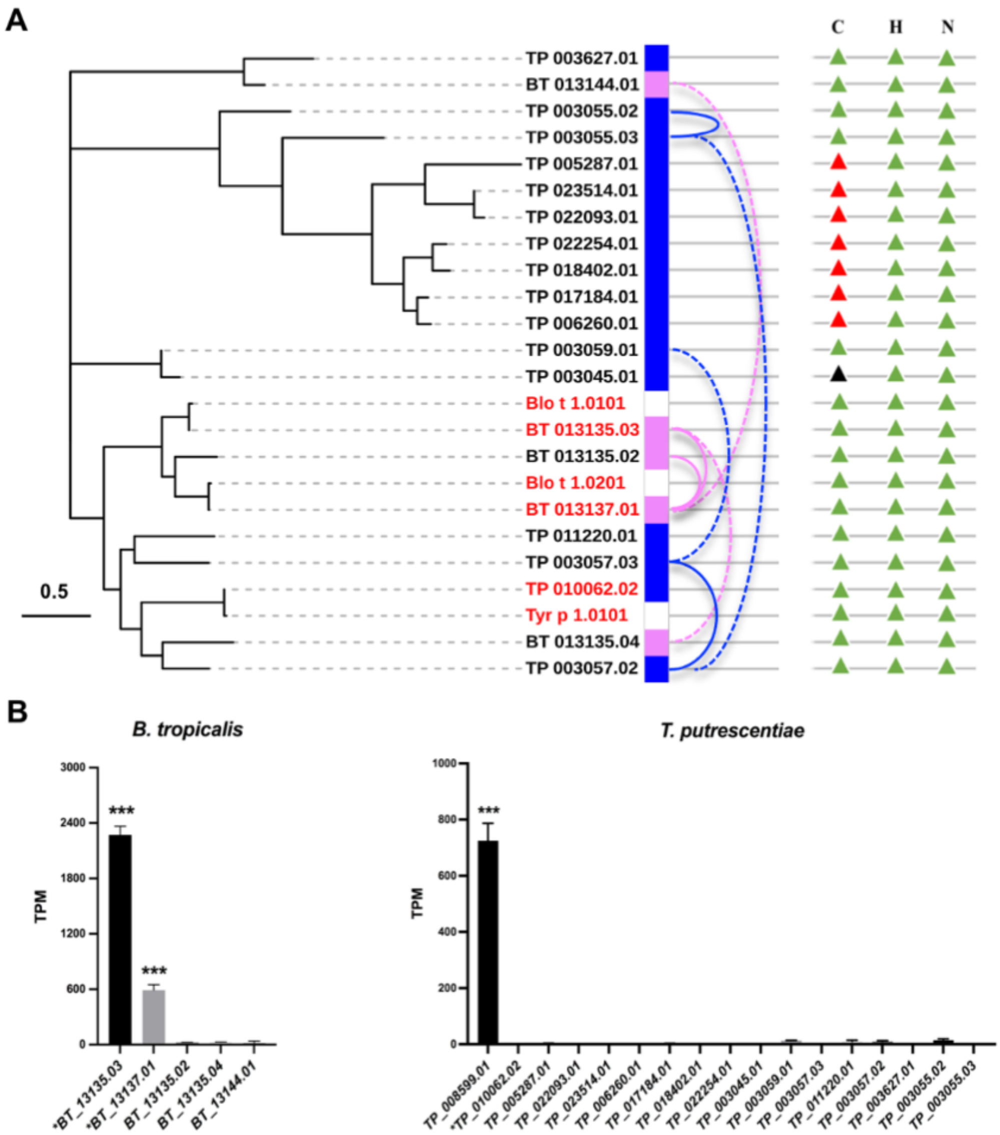

Supplementary Figure 9 Phylogenetic analysis and catalytic triad alignment of cysteine proteases in A1 cluster

(A) The phylogenetic analysis of D2 cluster genes, with the T/PAGs connected by solid curved lines and dashed curved lines, respectively. Blo t 1 and Tyr p 1 were marked as red in label. The catalytic triads (C-H-N) of D2 cluster genes were aligned, with active sites, site-mutated sites and deleted sites marked in green triangles, red triangles, and black triangles, respectively. “BT” as *B. tropicalis,* “TP” as *T. putrescentiae*. **(B)** The transcript expression levels of cysteine proteases were quantified as transcripts per million (TPM) using two adult mite transcriptome data of *B. tropicalis* and *T. putrescentiae*, respectively. Blo t 1 (BT_13135.03, BT_13137.01) and Tyr p 1 (TP_010062.02) were marked with asterisk. Data was shown as mean ± SD. Statistical significance was determined by one-way ANOVA, followed by Dunnett’s multiple comparison test. *** $P < 0.001$.

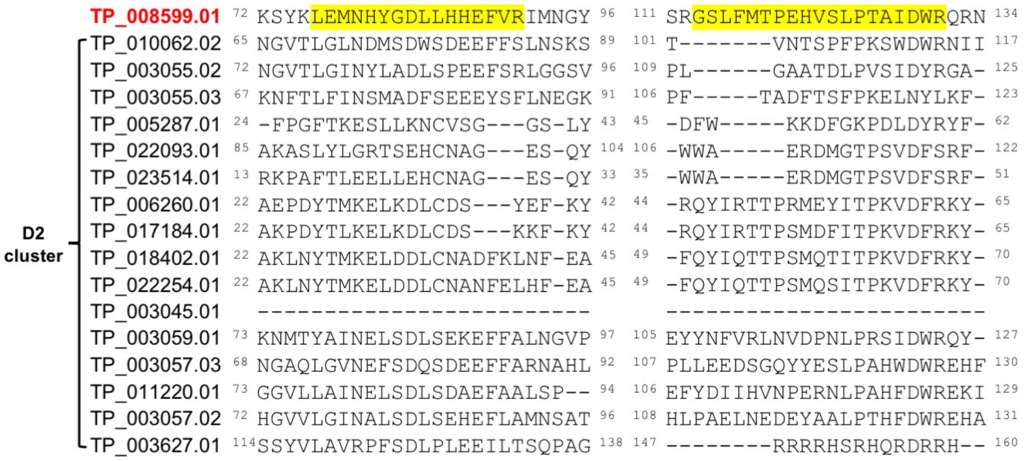

Supplementary Figure 10 The alignment of cysteine proteases from *T. putrescentiae*

The peptide sequences of IgE-binding proteins in *T. putrescentiae*-sensitized patient sera identified by MALDI-TOF MS (highlighted in yellow) were aligned to cysteine proteases of *T. putrescentiae*. The matched gene (TP_008599.01) was marked as red in label.

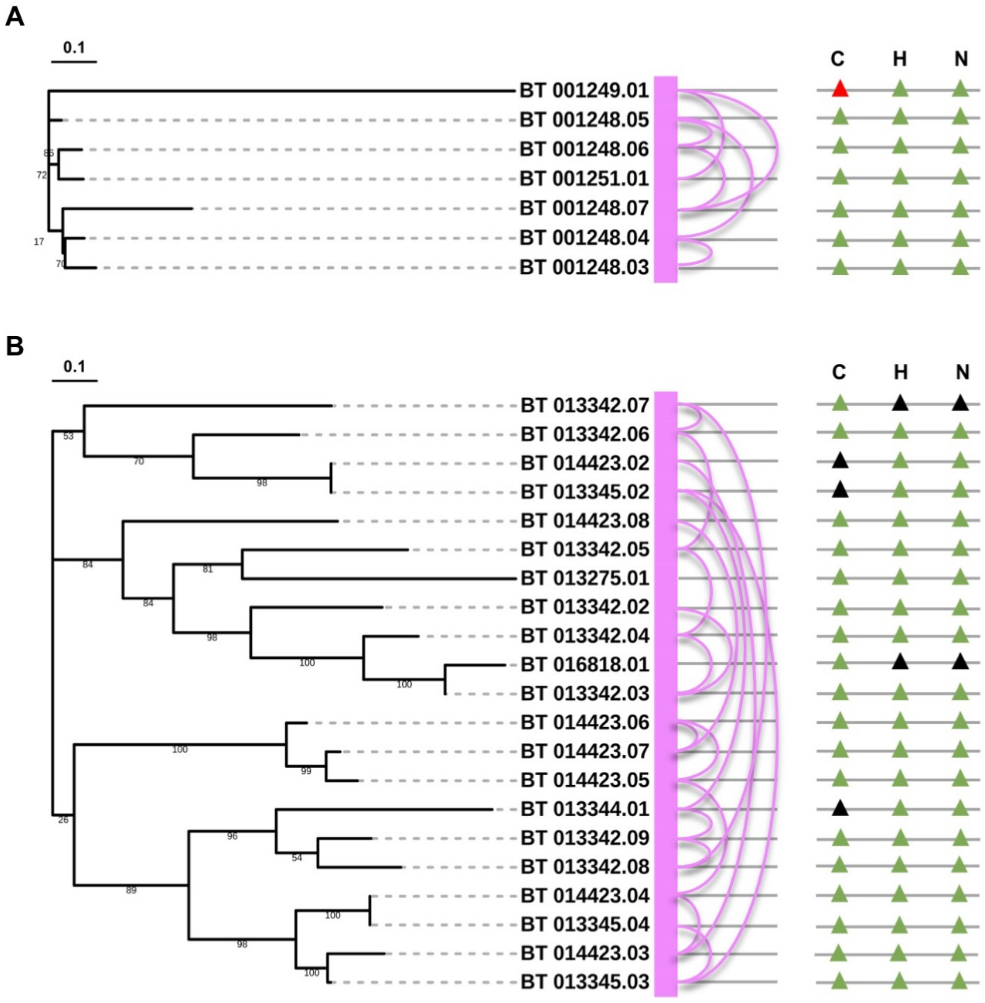

Supplementary Figure 11 Phylogenetic analysis and catalytic triad alignment of cysteine proteases in *B. tropicalis*

The phylogenetic analysis and catalytic triad alignment of **(A)** C1-1 cluster genes and (**B)** C5-1 cluster genes from *B. tropicalis*. The TAGs were connected by solid curved lines. The catalytic triads (C-H-N) of C1-1 and C5-1 cluster genes were aligned, with active sites, site-mutated sites and deleted sites marked in green triangles, red triangles and black triangles, respectively.

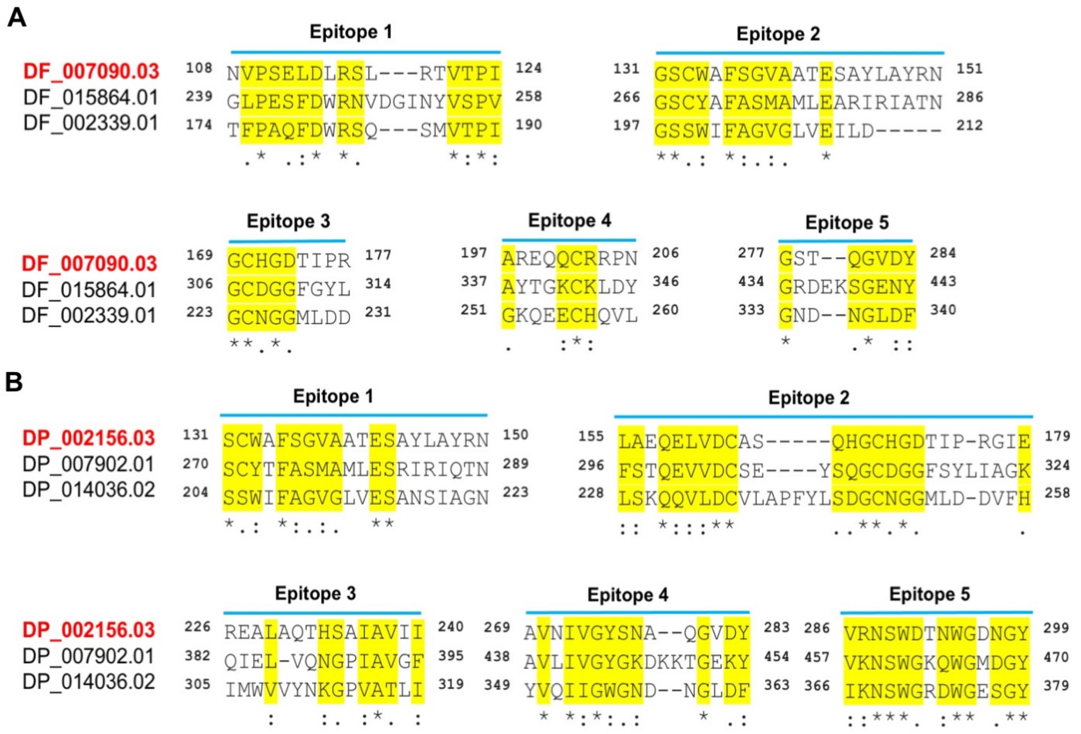

Supplementary Figure 12 The epitope mapping of cysteine proteases from HDMs in A2 and C3-1 cluster

The linear IgE epitope sequences of Der f 1 and Der p 1 were retrieved from The Immune Epitope Database (IEDB) and aligned to the cysteine proteases of **(A)** *D. farinae* and **(B)** *D. pteronyssinus* in A2 and C3-1 cluster. Der f 1 (DF_007090.03) and Der p 1 (DP_002156.03) were marked as red in label. The conserved site was denoted as " * ", conservative mutation as " : ", semi-conservative mutation as " . ". The sequences that are conserved, conservative mutated or semi-conservative mutated were highlighted in yellow.

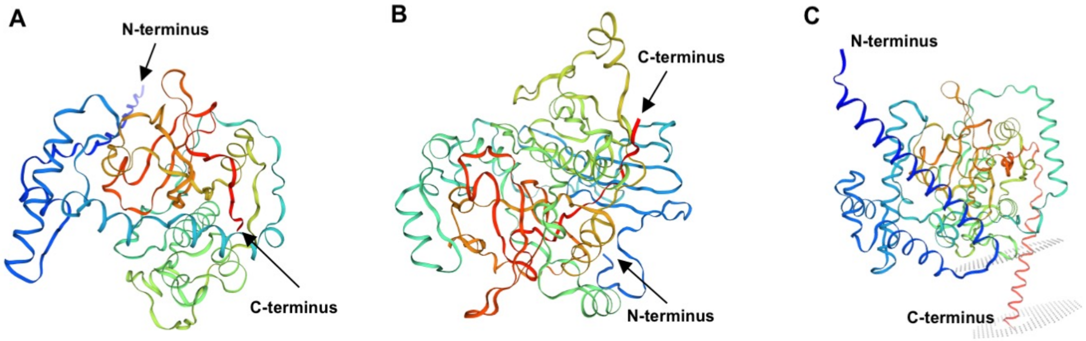

Supplementary Figure 13 The protein structure of cysteine proteases

The 3D structure of three cysteine proteases, **(A)** DP_002156.02, **(B)** DP_007902.01 and **(C)** DP_014036.02. The protein structures were predicted by SWISS-MODEL server using homology modeling.
